# Cuproptosis status affects treatment options about immunotherapy and targeted therapy for patients with kidney renal clear cell carcinoma

**DOI:** 10.3389/fimmu.2022.954440

**Published:** 2022-08-19

**Authors:** Ganghua Zhang, Xinyu Chen, Jianing Fang, Panpan Tai, Aiyan Chen, Ke Cao

**Affiliations:** Department of Oncology, Third Xiangya Hospital, Central South University, Changsha, China

**Keywords:** kidney renal clear cell carcinoma, cuproptosis, immune cell infiltration, immunotherapy, targeted therapy, prognosis

## Abstract

The development of immunotherapy has changed the treatment landscape of advanced kidney renal clear cell carcinoma (KIRC), offering patients more treatment options. Cuproptosis, a novel cell death mode dependent on copper ions and mitochondrial respiration has not yet been studied in KIRC. We assembled a comprehensive cohort of The Cancer Genome Atlas (TCGA)-KIRC and GSE29609, performed cluster analysis for typing twice using seven cuproptosis-promoting genes (CPGs) as a starting point, and assessed the differences in biological and clinicopathological characteristics between different subtypes. Furthermore, we explored the tumor immune infiltration landscape in KIRC using ESTIMATE and single-sample gene set enrichment analysis (ssGSEA) and the potential molecular mechanisms of cuproptosis in KIRC using enrichment analysis. We constructed a cuproptosis score (CUS) using the Boruta algorithm combined with principal component analysis. We evaluated the impact of CUS on prognosis, targeted therapy, and immunotherapy in patients with KIRC using survival analysis, the predictions from the Cancer Immunome Atlas database, and targeted drug susceptibility analysis. We found that patients with high CUS levels show poor prognosis and efficacy against all four immune checkpoint inhibitors, and their immunosuppression may depend on *TGFB1*. However, the high-CUS group showed higher sensitivity to sunitinib, axitinib, and elesclomol. Sunitinib monotherapy may reverse the poor prognosis and result in higher progression free survival. Then, we identified two potential CPGs and verified their differential expression between the KIRC and the normal samples. Finally, we explored the effect of the key gene *FDX1* on the proliferation of KIRC cells and confirmed the presence of cuproptosis in KIRC cells. We developed a targeted therapy and immunotherapy strategy for advanced KIRC based on CUS. Our findings provide new insights into the relationship among cuproptosis, metabolism, and immunity in KIRC.

## Introduction

Renal cell carcinoma (RCC) is one of the most common malignancies of the urinary system, and its most common subtype is kidney renal clear cell carcinoma (KIRC), which accounts for approximately 75% of all RCC cases (1). Currently, early resection is considered the basic treatment for patients with KIRC (2); however, up to 40% of patients develop metastases after primary surgical treatment for local RCC, resulting in poor prognosis (3). Furthermore, randomized controlled clinical studies have shown that postoperative adjuvant cytokine therapy, radiotherapy and chemotherapy have little impact on reducing recurrence and metastasis rates (4, 5). Therefore, once KIRC is metastatic or unresectable, the treatment options become very limited.

The rapid development of targeted therapy and immunotherapy has been a turning point in treating patients with metastatic or unresectable KIRC. These two treatments and their combinations have become the required options for these patients. Initially, targeted therapy was shown to substantially prolong survival in advanced KIRC (6, 7). Multitarget drugs, such as sunitinib and sorafenib, have become the first choice for patients with metastatic or unresectable KIRC (8). In recent years, researchers have found that targeted therapy combined with immunotherapy has beneficial treatment effects and good prospects in patients with advanced KIRC, and has shown a trend of gradually replacing targeted therapy alone (9). However, many patients do not benefit from the combined therapy because of the overlapping drug toxicity that affects their quality of life (10). Therefore, new molecular phenotypes should be established to divide the population more finely for the individualized selection of effective immunotherapy drugs or targeted drugs for patients with advanced or unresectable KIRC.

Copper (Cu) ion is a double-edged sword in the life activities of cells: on the one hand, Cu ions are key co-factors for many enzymes, such as cytochrome c oxidase, which relies on Cu ions to complete cellular respiration (11). On the other hand, excess Cu ions induce cuproptosis in cells (12). Cuproptosis is an emerging form of programmed cell death, dependent on intracellular copper accumulation, which is distinct from the known forms of cell death, such as ferroptosis, pyroptosis, and apoptosis. Human mitochondrial ferredoxin 1 (*FDX1*) positively regulates lipoylated enzymes, and Cu ions directly bind to lipoylated components in the tricarboxylic acid cycle (TCA) pathway, resulting in abnormal aggregation of lipoylated proteins and loss of iron-sulfur cluster proteins and proteotoxic stress response. This eventually leads to cuproptosis of cells (13). Cu ions, protein lipoylation, and mitochondrial respiration are important determinants of cuproptosis. Therefore, in cancers that express a large number of lipoylated mitochondrial proteins and have a high degree of respiration, the use of metal carriers to transport Cu ions and activate cuproptosis can kill cancer cells with this metabolic feature. This approach could potentially become a new cancer treatment (14). KIRC cells show the classic Warburg effect as the main metabolic feature (15). Since they do not participate in the mitochondrial respiratory pathway, we hypothesized that cuproptosis would be inhibited in KIRC; thus, it is important to promote cuproptosis in KIRC. In addition, intratumoral copper levels affect the expression of programmed death ligand 1 (PD-L1) in cancer cells, and Cu regulates a key signaling pathway that mediates PD-L1-driven cancer immune evasion (16). However, no reports have suggested a correlation between cuproptosis and immunotherapy, and no studies are available on the effect of cuproptosis on KIRC.

In this study, we selected seven genes that promote cuproptosis as starting points for typing. We comprehensively used the KIRC cohort of The Cancer Genome Atlas (TCGA) and Gene Expression Omnibus (GEO) databases to establish novel molecular typing and explored the intratumoral immune infiltration landscape of KIRC using ESTIMATE and single-sample gene set enrichment analysis (ssGSEA) algorithms. We used a novel scoring model, the cuproptosis score (CUS), to predict the prognosis and the efficacy of targeted therapy and immunotherapy in patients with KIRC and explored specific targets and drugs. Our results provide a new and detailed strategy for individualized targeted therapy and immunotherapy in patients with advanced or unresectable KIRC.

## Materials and methods

### Exploration of the genetics and biological significance of cuproptosis-promoting genes in KIRC

P. Tsvetkov et al. identified seven cuproptosis-promoting genes (CPGs): *FDX1*, *LIAS*, *LIPT1*, *DLD*, *DLAT*, *PDHA1* and *PDHB* (13). The Gene Set Cancer Analysis (GSCA) database (http://bioinfo.life.hust.edu.cn/GSCA/#/) was used to analyze differential mRNA expression, single nucleotide variation (SNV), copy number variation (CNV), and methylation of seven CPGs (17). A network of seven CPGs was drawn using the “igraph” R package. Attributes in the network were determined using intergene correlations and univariate Cox regression analysis.

### Data collection and processing of KIRC comprehensive cohort

The TCGA-KIRC cohort containing 534 KIRC samples from the TCGA database (https://tcga-data.nci.nih.gov/tcga/) and the GSE29609 cohort (platform GPL1708) containing 39 KIRC samples from the GEO database (http://www.ncbi.nlm.nih.gov/geo/) were used for data merging. Normalized matrix files and clinical data were downloaded from the GEO database; RNA sequencing data (fragments per kilobase million, FPKM values) of gene expression and clinical data were obtained from TCGA. The FPKM values were then converted to transcripts per kilobase million (TPM) values for further analysis, “ComBat” from the “SVA” R package was used to eliminate the batch effects (18), and principal component analysis (PCA) was used to eliminate the batch effects. Samples without complete survival data were excluded. Finally, we obtained a comprehensive KIRC cohort containing 537 samples and 14074 genes.

### First unsupervised clustering based on seven CPGs

We used the “ConsensusClusterPlus” R package for unsupervised clustering and classification based on seven CPGs (19), using agglomerative pam clustering with the Euclidean distance and resampling 80% of the samples for 50 repetitions. We then used survival analysis to compare the differences in overall survival (OS) between different subtypes, box plots to compare the expression of seven CPGs between different subtypes, and used the “pheatmap” R package to draw a cluster heatmap to show the relationship between the expression of seven CPGs, clinicopathological features, and classification.

### Gene set variation analysis

We downloaded the data of the HALLMARK pathway, the Kyoto Encyclopedia of Genes and Genomes (KEGG) pathway, and the Reactome pathway from the Molecular Signatures Database (MsigDB, http://software.broadinstitute.org/gsea/msigdb/), and acquired “h.all.v7.5.1. symbols.gmt,” “c2.cp.kegg.v7.5.1. symbols.gmt,” and “c2.cp.reactome.v7.5.1. symbols.gmt” as reference gene sets (20). Then, we used the “GSVA” R package to perform Gene Set Variation Analysis (GSVA) for different subtypes and drew a heatmap to display the analysis results.

### Construction of KIRC immune infiltration landscape

The “ESTIMATE” R package was used to calculate StromalScore, ImmuneScore, and ESTIMATEScore. StromalScore and ImmuneScore represent the abundance of stromal and immune components, respectively, whereas ESTIMATEScore is the sum of StromalScore and ImmuneScore, which is negatively correlated with tumor purity (21). The “GSVA” R package was then used for ssGSEA to calculate the enrichment score that represents the relative infiltrating abundance of each immune cell (22).

### Screening of differentially expressed genes (DEGs) and enrichment analysis

The “limma” R package (23) was used to screen for differentially expressed genes (DEGs) between different subtypes with |logFoldChange| >1 and p <0.05. KEGG and Gene Ontology (GO) functional enrichment analyses were implemented using the “clusterProfiler” R package (24), and adjusted p-value <0.05 represented statistically significant results.

### Secondary unsupervised clustering based on cuproptosis subtypes related genes

We screened the DEGs with p <0.05 using univariate Cox regression analysis and named them cuproptosis subtype-related genes (CSRGs). The “forestplot” R package was used to draw a forest plot of the results. Then, secondary unsupervised clustering classification was performed based on CSRGs, with the same specific clustering parameters. Subsequently, we used survival analysis to compare the differences in OS between different subtypes, used box plots to compare CSRGs expressions between different subtypes, and drew a cluster heatmap to show the relationship among CSRGs expression, clinicopathological features, and classification.

### Calculation of cuproptosis score (CUS)

According to the positive and negative relationships between the CSRGs and the cluster signature, the CSRGs were divided into two groups, namely sigC1 and sigC2. Then, the “clusterProfiler” R package was used for gene annotation. We then used the Boruta algorithm (25)combined with PCA to reduce the dimensionality of the CSRGs subgroups and calculated the CUS for each sample. The KIRC comprehensive cohort was divided into the high- and low-CUS groups based on the optimal cutoff value. The CUS of each KIRC sample was calculated using the following formula:


CUS=∑PsigC1−∑PsigC2

### Prognosis and immune exploration based on CUS grouping

We used the “survival” and “survminer” R packages to perform survival analysis to compare the differences in OS between the high- and low-CUS groups, and used the “ggalluvial” R package to draw Sankey diagrams to visualize the correspondence among CUS groups, different subtypes, and prognosis. Box plots were used to compare the differences in the CUS of different subtypes. ssGSEA was used to quantify the infiltration abundance of immune cells, and the relationship between CUS and immune cell infiltration levels was displayed using a correlation heat map.

### Clinical subgroup analysis based on CUS grouping

We selected “survival status,” “histological grade,” “T stage,” “N stage,” “M stage” and “clinical stage” as clinical subgroup characteristics, and drew box plots to show the differences in the CUS between different clinical characteristics. A stacked histogram was drawn to show the proportion of each clinical characteristic in the high- and low-CUS groups.

### Comparison of immune targets and prediction of immunotherapy efficacy

We used the “limma” R package to compare the differences in the gene expression of several common immune targets. Next, we downloaded the immunophenoscore (IPS) data of the TCGA-KIRC cohort from The Cancer Immunome Atlas (TCIA) database to explore the differences in the efficacy of the four immune checkpoint inhibitors (ICIs) between the high- and low-CUS groups (26), including ctla4_pos_pd1_pos, ctla4_neg_pd1_pos, ctla4_pos_pd1_neg, and ctla4_neg_pd1_neg.

### Analysis of targeted therapy based on CUS grouping

We estimated the half maximal inhibitory concentration (IC50) using the “pRRophetic” R package to predict the sensitivity of the high- and low-CUS groups to 138 targeted drugs. We selected eight commonly used targeted drugs for advanced KIRC (sunitinib, axitinib, sorafenib, erlotinib, lapatinib, gefitinib, pazopanib, and temsirolimus) and a cuproptosis-targeting drug (elesclomol) for key observation (27). Subsequently, we downloaded the gene expression profile and clinical data of the sunitinib monotherapy cohort in the NCT02684006 clinical trial from the supplementary material of PMID:32895571 (28). We used differential analysis to explore the relationship between CUS groups and progression and compared the differences in progression-free survival (PFS) between the high- and low-CUS groups *via* survival analysis. The significance of the difference in comparing the progression rates of different CUS groups was achieved by the chi-square test.

### Mining seven CPGs-related targeted drugs

We calculated the correlation between the mRNA expression of seven CPGs and drug IC50 values *via* Pearson’s correlation analysis of the “GDSC drug” and “CTRP drug” modules in the GSCA database. The p-value was adjusted using the false discovery rates (FDR).

### Screening and validation of potential CPGs and *FDX1*

Hazard ratio (HR) and p values of univariate COX regression analysis were used to identify potential CPGs. Potential CPGs were screened using p < 0.001 and 1-HR > 0.4 as the inclusion criteria. Then, a comprehensive analysis was performed integrating TCGA and the Genotype Tissue Expression (GTEx) (https://commonfund.nih.gov/GTEx/) databases (29) through the “Expression DIY” module of the Gene Expression Profiling Interactive Analysis (GEPIA, http://gepia.cancer-pku.cn/) website (30). |Log2FC| > 1 and p <0.01 were set as the cutoff values. Additionally, immunohistochemical (IHC) staining results of the three genes between normal renal tubular epithelial and KIRC tissues at the protein level were obtained from Human Protein Atlas (HPA, https://www.proteinatlas.org/) database.

### Cell culture and transfection

Human renal tubular epithelial cells (HK-2) and KIRC cells (Caki-1 and 786-O) were obtained from the American Type Culture Collection (ATCC, Manassas, VA, USA). All cells were cultured in RPMI 1640 medium (Hyclone, Logan, UT, USA) supplemented with 15% fetal bovine serum (Gibco, Grand Island, NY, USA) and 1% penicillin-streptomycin (Hyclone). Small interfering RNAs (siRNAs) targeting FDX1 were synthesized by GenePharma (Shanghai, China). siRNA-FDX1 and siRNA-control were cotransfected into Caki-1 cells using Lipofectamine 2000 (Invitrogen, Carlsbad, CA, USA). The primer sequences of siRNAs are listed in **Supplementary Table 2**.

### Quantitative reverse transcription polymerase chain reaction

Total RNA from the cultured cells was extracted using a Faster reagent (Invitrogen). The PrimeScript RT Reagent Kit (TaKaRa, Shiga, Japan) was used to reverse transcribe 1µg total RNA into cDNA, and the SYBR Green PCR Master Mix was used for quantitative reverse transcription polymerase chain reaction (qRT-PCR). Relative gene expression was calculated using equation 2^–ΔΔCT^, with GAPDH as an internal loading control. Visualization of qRT-PCR results and two samples unpaired t-test was performed using GraphPad Prism version 9.0.1 (GraphPad Software, San Diego, California USA, www.graphpad.com). All the primers used for qRT–PCR were synthesized by Tsingke Biotech (Tsingke, China). The primer sequences used are listed in **Supplementary Table 2**.

### Western blotting

The total protein in human renal tubular epithelial cells (HK-2) and KIRC cells (Caki-1 and 786-O) were extracted using radioimmunoprecipitation assay (RIPA) buffer (Beyotime, Shanghai, China), Protease Inhibitor Cocktail (Cwbio, CW2200) and Phosphatase Inhibitor Cocktail (Cwbio, CW2383) for 20 min at 4°C. Protein concentration was determined using a BCA protein assay kit (Beyotime, Shanghai, China). The protein samples were separated on an SDS-PAGE Loading Buffer (Cwbio, CW0027), and transferred onto polyvinylidene difluoride (PVDF) membranes (Millipore, IPVH00010). Subsequently, the membrane was blocked in Tris-buffered saline plus tween-20 (TBST; Servicebio, G0001) containing 5% nonfat powdered milk (Sangon Biotech, A600669) for 1 h. Anti-FDX1 (1:1000, A20895; Abclonal), anti-ACAT1 (1:1000; 16215-1-AP, Proteintech), Beta Tubulin (1:1000; 10094-1-AP, Proteintech) and GAPDH (1:1000; R24404, Zen-Bioscience) were used as primary antibodies, and the membrane was submerged in primary antibodies overnight at 4°C. Then, the membrane was washed with TBST 3 times and incubated with Goat Anti-Rabbit IgG-Horseradish Peroxidase (1:5000; Elabscience, E-AB-1003) for 1 h at room temperature. Immunoassay was performed by an enhanced chemiluminescence detection system (ECL; Biosharp, BL520A) combined with a Western blot system (Auragene). The expression of the target band relative to the loading control was quantified with integrated density by ImageJ software.

### Cell Counting Kit-8 (CCK-8) assay

The transfected Caki-1 cells were adjusted to 2000 cells/well (100 μL medium) in 96-well plates and cultured for the indicated days. Then, 10 μL of CCK-8 (BS350A, Biosharp Life Sciences) was added to the cells, and the cells were cultured at 37°C for 1.5 hours. Optical density was measured at 450 nm (OD_450nm_) using a microplate reader.

Caki-1 cells were transferred into 96-well plates at a density of 2500 cells/well (100 μL medium). After 24 h the cells were divided into 8 groups (n=3 per group), incubated with 100μL of fresh medium containing CuCl_2_ (100nM; RHAWN, R019783), Elesclomol (100nM; MedChemExpress, HY-12040), CuCl_2_ (100nM)& Elesclomol (100nM), CuCl_2_ (200nM), Elesclomol (200nM), CuCl_2_ (200nM)& Elesclomol (200nM) or control agents for indicated days. Briefly, 10 μL of CCK-8 was added and OD_450nm_ was measured.

### Ethynyl-2’-deoxyuridine (EdU) assay

Caki-1 cells were stained using BeyoClick™ EdU-555 Cell Proliferation Kit (Beyotime, Shanghai, China). To be specific, Caki-1 cells (1.0×10^5^ cells/well) were seeded in a 6-well plate, transfected with NC or si-FDX1, and cultured in an incubator at 37°C for 72 h. Then, Caki-1 cells were incubated with EdU for 2 h, fixed with 1 mL paraformaldehyde (4%) for 15 min, and permeabilized with 0.3% Triton X-100 (Beyotime) for 15 min. After that, the Caki-1 cells were incubated with 500µL of the click reaction mixture for 30 min in the dark, washed three times with PBS containing 3% BSA, and incubated with Hoechst 33342 for another 10 min. Finally, fluorescence microscopy was used for detection.

### Colony formation assay

The transfected cells were cultured up to the logarithmic growth phase and then counted and adjusted to 500 cells/well in 6-well plates. Cells were incubated for 2 weeks at 37°C, 5% CO_2_. After being washed with PBS twice, the cells were fixed with paraformaldehyde (4%) for 15 min and stained with crystal violet buffer (Solarbio, Beijing, China) for 30 min. The clone was counted if the number of cells in the clone was at least 50 under a microscope.

### Statistical analysis

All analyses were performed using R version 4.1.1. Unless otherwise specified, Pearson’s correlation coefficient was used for correlation analysis in this study. For comparison between the two groups in the bioinformatics analysis section, the Wilcoxon test was used for difference analysis. For comparison between the two groups in the experimental section, the Students’ t-test was used for difference analysis. Two-way ANOVA was used for difference comparison in CCK8 assay. For comparisons between more than two groups, the Kruskal-Wallis test was used for the difference analysis. Kaplan-Meier survival analysis and log-rank tests were used to compare the survival of the different groups of patients. For all statistical analyses, a two-tailed p <0.05 was considered statistically significant.

## Results

### Genetic, transcriptional and post-transcriptional alterations of CPGs in KIRC

**Figure 1:**
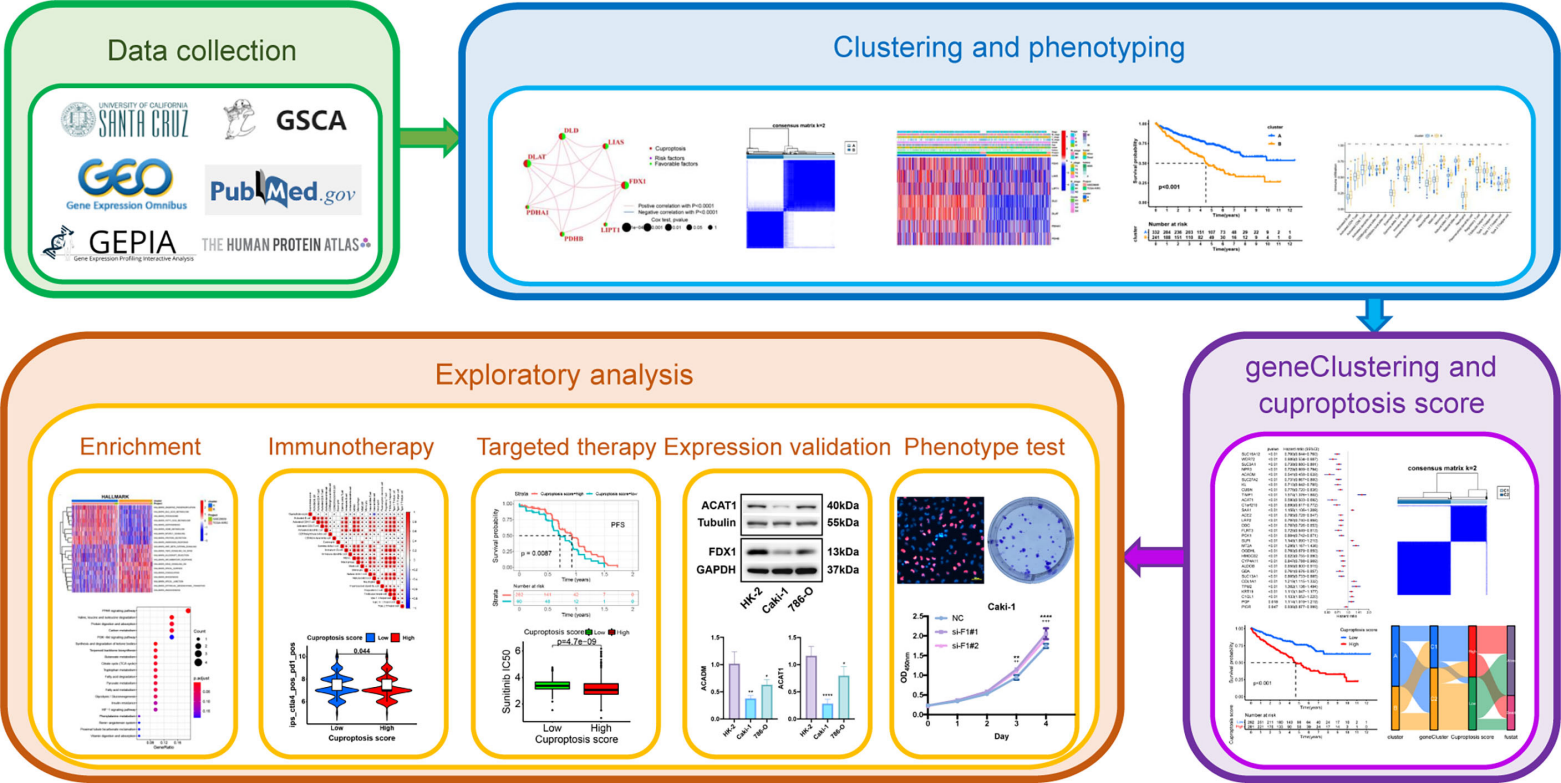
The flowchat of this study.

The workflow of this study is illustrated in **Figure 1**. We performed different levels of analysis of the seven CPGs using the GSCA database. At the mRNA level, *DLAT*, *DLD*, *FDX1*, *PDHB*, and *PDHA1* showed low expression in KIRC compared to the normal samples (FDR <0.05, **Supplementary Figure 1A**). The SNV frequencies of the six CPGs are shown in the form of a heat map (no data are available for *FDX1*), with DLD having the highest SNV frequency (**Supplementary Figure 1B**). Seven CPGs showed large differences in CNV types (including heterozygous amplification, homozygous amplification, heterozygous deletion, and homozygous deletion) and proportions, and *PDHB* had a very large proportion of heterozygous deletions, whereas *DLD* had only heterozygous amplification (**Supplementary Figure 1C**). The CNV of CPGs was positively correlated with their mRNA expression, especially that of *PDHB* (**Supplementary Figure 1D**). Conversely, methylation levels of CPGs were negatively correlated with mRNA expression (**Supplementary Figure 1E**). However, the methylation of CPGs was not significantly different between KIRC and normal samples (no data available for *FDX1*, **Supplementary Figure 1F**).

### Construction of comprehensive KIRC cohort and CPGs network

**Figure 2:**
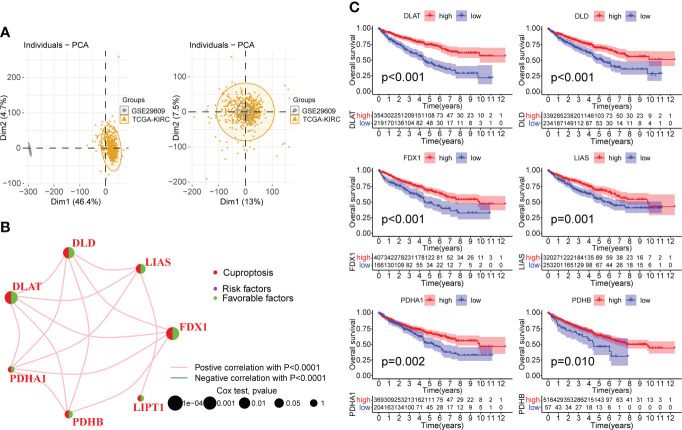
Correlation analysis and survival analysis of CPGs in KIRC. **(A)** Fusion of TCGA-KIRC and GSE29609 cohort data and removal of batch effects. The left plot shows PCA before removing batch effects, and the right plot shows PCA post removing batch effects. **(B)** Correlation network of seven CPGs. The line represents a correlation between genes, the sphere represents the COX test of each gene. **(C)** Kaplan-Meier survival analysis of CPGs in KIRC (OS, Log-rank test, p < 0.05). CPG, cuproptosis-promoting gene.

We merged the TCGA-KIRC and GSE29609 datasets and removed the batch effects to obtain a comprehensive cohort of 537 samples and 14,074 genes. PCA showed that batch effects were effectively eliminated (**Figure 2A**). The baseline data on the clinical characteristics of the comprehensive cohort are presented in **Table 1**. The network of seven CPGs showed the results of the correlation analysis and Cox regression analysis; seven CPGs had significant positive correlations (p <0.0001) and were protective factors for KIRC (HR < 1, **Figure 2B**). Finally, the Kaplan-Meier survival analysis suggested that six CPGs significantly affected the prognosis of patients with KIRC (p <0.05, **Figure 2C**).

**Table 1:** Baseline Data Sheet for the Comprehensive Cohort of TCGA-KIRC and GSE29609.

Characteristic	levels	N (%)
Age	>60 years old	291 (50.8%)
	≤60 years old	282 (49.2%)
Grade	G1	15 (2.7%)
	G2	243 (43%)
	G3	217 (38.4%)
	G4	90 (15.9%)
T_stage	T1	285 (49.7%)
	T2	75 (13.1%)
	T3	201 (35.1%)
	T4	12 (2.1%)
N_stage	N0	271 (91.9%)
	N1	21 (7.1%)
	N2	3 (1%)
M_stage	M0	449 (83%)
	M1	92 (17%)
Stage	I	268 (50.5%)
	II	58 (10.9%)
	III	123 (23.2%)
	IV	82 (15.4%)

### Identification and evaluation of subtypes based on seven CPGs

**Figure 3:**
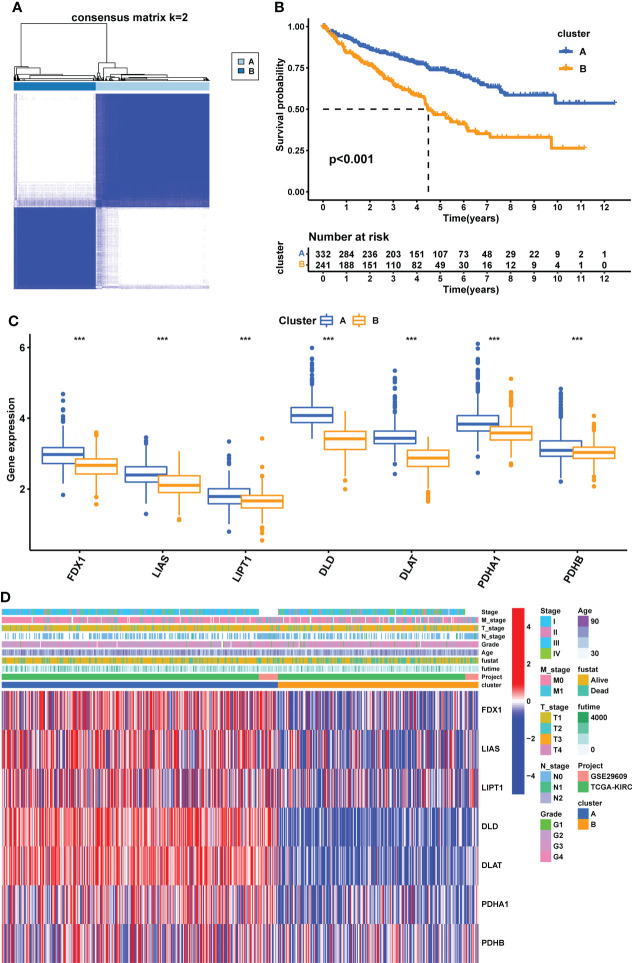
Construction of CPG subtypes and exploration about clinical and biological features of subtypes. **(A)** consensus matrix that divides all KIRC samples into two clusters (k=2). **(B)** Kaplan–Meier curves for the two subtypes of KIRC patients (OS, Log-rank test, p < 0.001). **(C)** Expression differences of seven CPGs between the two subtypes. **(D)** Heatmap of the distribution of clinicopathological features and CPG expression between two different subtypes. CPG, cuproptosis-promoting gene; ***p < 0.001.

We performed unsupervised clustering and classification based on seven CPGs. The best classification effect could be obtained when the patients were divided into Clusters A and B (**Figure 3A**). The clustering results are shown in **Supplementary Figures 2A–C**. There was a significant difference in OS between the two subtypes, with cluster A having a better prognosis than cluster B (p <0.001, **Figure 3B**). Seven CPGs showed higher expression levels in cluster A (p <0.001, **Figure 3C**). We showed the clinicopathological features of the two subtypes and the expression distribution of the seven CPGs using a heat map (**Figure 3D**). We used GSVA to compare the enrichment pathways of the two subtypes from the three sets of the HALLMARK pathway (**Supplementary Figure 3A**), KEGG pathway (**Supplementary Figure 3B**), and Reactome pathway (**Supplementary Figure 3C**), and detected significant differences between the two subtypes, mainly in multiple metabolic pathways.

**Figure 4:**
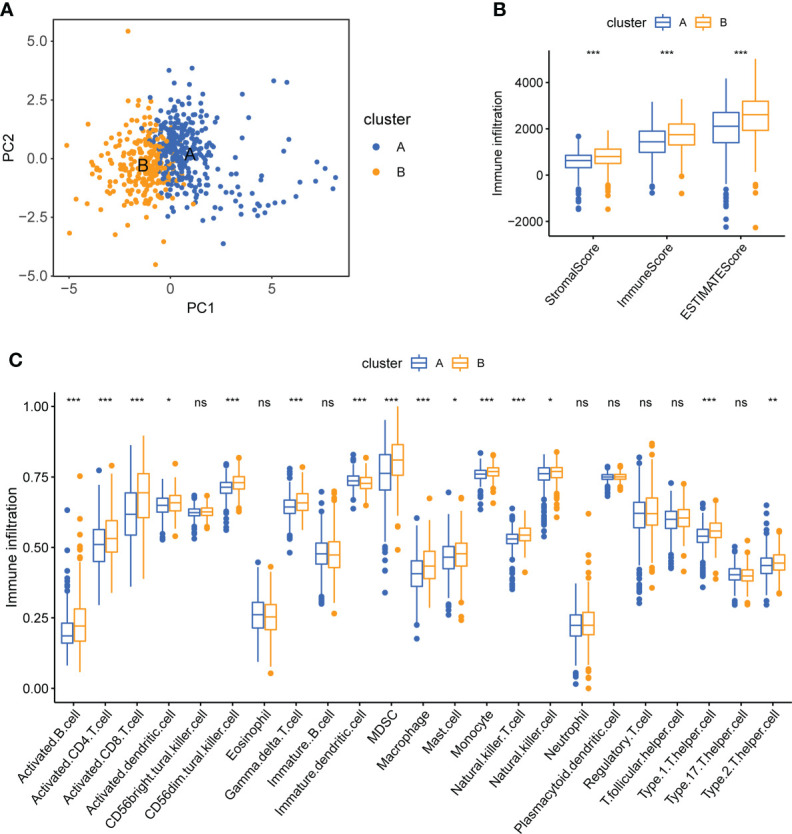
Tumor immune microenvironment analysis of two CPG subtypes. **(A)** PCA shows a significant difference in transcriptomes between the two subtypes. **(B)** Correlations between the two CPG subtypes and TME score. **(C)** The abundance of 23 kinds of infiltrating immune cells was evaluated by ssGSEA in the two CPG subtypes. CPG, cuproptosis-promoting gene; ns, no significant difference, *p < 0.05; **p < 0.01; and ***p < 0.001.

Patients in different clusters showed feature distinguishability based on PCA (**Figure 4A**). Next, we used ESTIMATE to quantify the infiltration characteristics of the tumor microenvironment in patients with KIRC and observed that Cluster B had higher StromalScore, ImmuneScore, and ESTIMATEScore than Cluster A (p <0.001, **Figure 4B**). We used ssGSEA to quantify the infiltrating abundance of 23 immune cells and explored the differential patterns of the immune-infiltrating landscape of the two subtypes. The infiltration levels of activated B cells, CD4 T cells, CD8 T cells, dendritic cells, CD56^dim^ structural killer cells, gamma delta T cells, myeloid-derived suppressor cells (MDSC), macrophages, mast cells, monocytes, natural killer T cells, natural killer cells, type 1 T helper cells, and type 2 T helper cells were significantly higher in cluster B than in cluster A (p <0.05, **Figure 4C**). These results suggest that Cluster B has a higher level of stromal and immune cell infiltration than Cluster A.

### Identification of CSRGs and the secondary clustering

**Figure 5:**
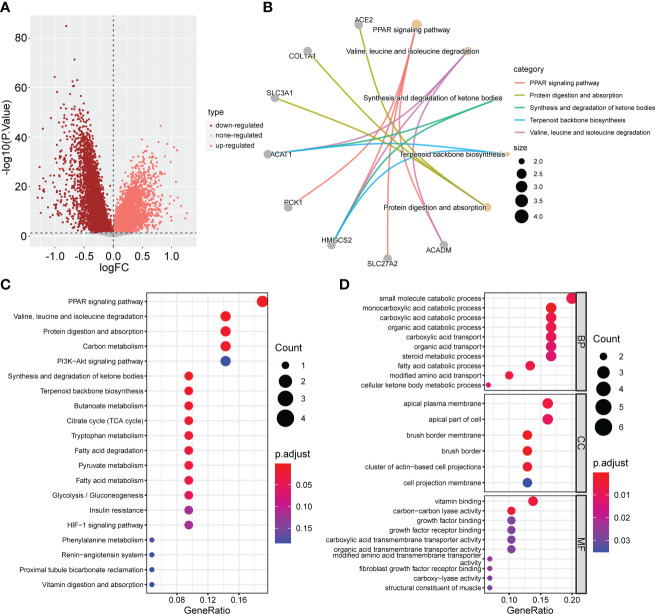
Screening and enrichment analysis of DEGs between the two CPG subtypes. **(A)** Volcano plot about Difference analysis between the two CPG subtypes. **(B)** The network diagram shows the correspondence between the KEGG top five pathways and related genes. **(C)** KEGG enrichment analysis of DEGs among two CPG subtypes. **(D)** GO enrichment analysis of DEGs among two CPG subtypes. DEG, differentially expressed gene; CPG, cuproptosis-promoting gene.

To further explore the potential biological behavior of each cuproptosis subtype, we performed a differential analysis of the two cuproptosis subtypes. The DEGs are shown using volcano plots (p <0.05, **Figure 5A**). We then screened DEGs with |logFoldChange| >1 and p <0.05, and identified 31 cuproptosis-related DEGs. KEGG (**Figure 5C**) and GO (**Figure 5D**) enrichment analyses were performed on these DEGs, and the top five pathways based on the adjusted p-value in KEGG analysis and their relationship networks with related genes were displayed (p <0.05, **Figure 5B**). Several pathways were related to mitochondrial metabolism, including the peroxisome proliferator-activated receptor (PPAR) signaling pathway, carbon metabolism, citrate cycle (TCA cycle), fatty acid degradation, and the PI3K-Akt signaling pathway.

**Figure 6:**
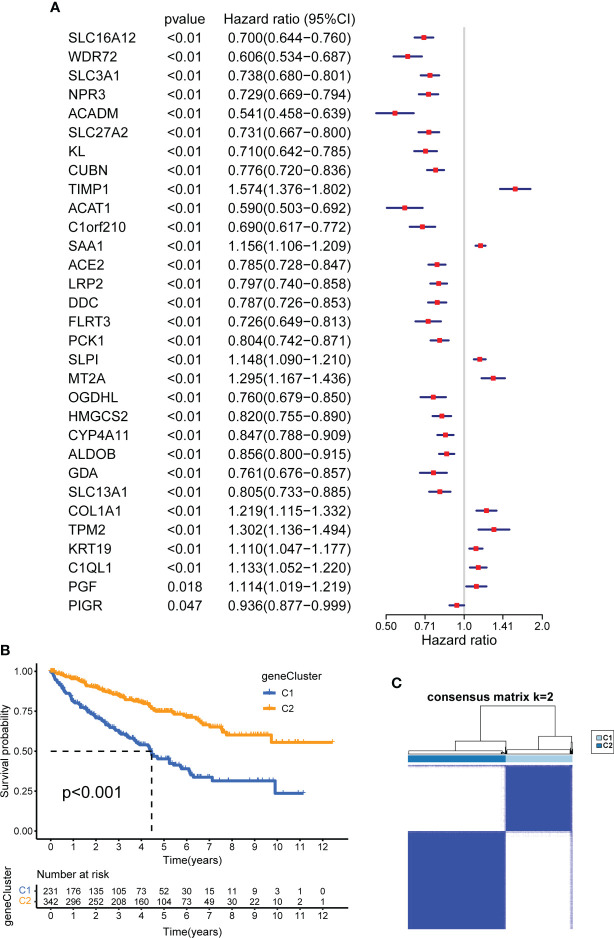
Construction of CSRG gene subtypes and prognostic analysis. **(A)** Univariate COX regression analysis of 31 DEGs to screen CSRGs. **(B)** Kaplan–Meier curves for the two gene subtypes (OS, Log-rank test, p < 0.001). **(C)** consensus matrix that divides all samples into two geneclusters (k=2). CSRG, cuproptosis subtypes related gene; DEG, differentially expressed gene.

To identify cuproptosis-related genes with prognostic significance for KIRC, we performed a univariate Cox regression analysis on 31 cuproptosis-related DEGs; the results are shown in **Supplementary Table 1**. All 31 DEGs had prognostic significance and were identified as CSRGs (p <0.05, **Figure 6A**). Based on 31 CSRGs, we performed secondary clustering and identified two subtypes: gene clusters C1 and C2 (**Figure 6C**). The clustering results are shown in **Supplementary Figures 2D–F**. The plots of the Kaplan-Meier survival curves of C1 and C2 showed that C2 had a better prognosis than C1 (p <0.001, **Figure 6B**). The 31 CSRGs were significantly differentially expressed between C1 and C2 (p <0.001, **Supplementary Figure 4A**). We used a heatmap to show the clinicopathological characteristics of the two subtypes and the expression distribution of the 31 CSRGs (**Supplementary Figure 4B**). According to the positive and negative relationships between DEGs and cluster features, CSRGs were divided into two groups: sigC1 and sigC2. The genes in the sigC1 group were highly expressed in C2 and lowly expressed in C1, whereas the opposite was true for the genes in the sigC2 group.

### Calculation of CUS and classification of patients with KIRC

**Figure 7:**
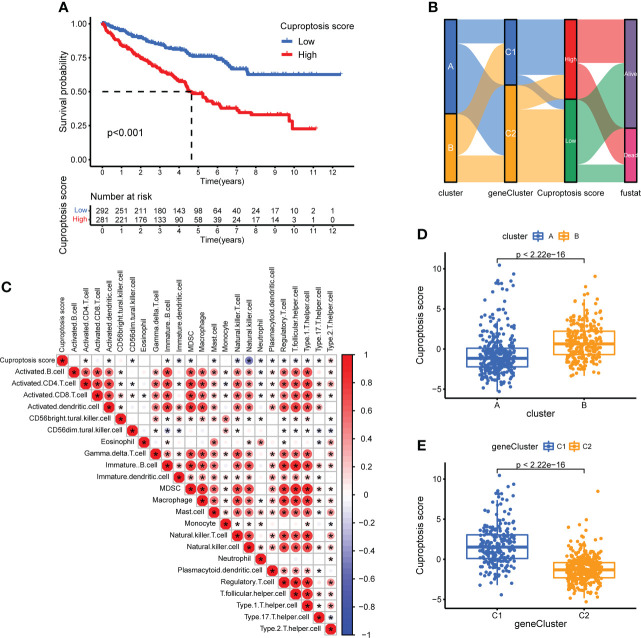
Construction of CUS and grouping based on score. **(A)** Kaplan–Meier survival analysis between the high- and low-CUS groups (OS, Log-rank test, p < 0.001). **(B)** Sankey diagram of subtype distributions in groups with different CUSs and survival outcomes. **(C)** The correlation matrix of cuproptosis and all 22 infiltrating immune cells. Red means positive correlation, whereas blue means negative correlation. p < 0.05 was the cut-off. **(D)** Differences in CUS levels between the two CPG subtypes. **(E)** Differences in CUS levels between the two CSRG gene subtypes. CUS, cuproptosis score; CPG, cuproptosis-promoting gene; CSRG, cuproptosis subtypes related gene; *p < 0.05.

To quantify the cuproptosis status to predict the clinical characteristics and treatment outcomes of the patients, we calculated the CUS for each sample using the Boruta algorithm combined with PCA based on two gene sets, sigC1 and sigC2. According to the best cutoff value “-0.4318927,” the samples of the comprehensive cohort were divided into the high- and low-CUS groups. The results of the Kaplan-Meier survival analysis revealed that patients in the high-CUS group had a poorer prognosis (**Figure 7A**). The Sankey diagram shows the corresponding relationship among the CUS, classification, and prognosis (**Figure 7B**). Patients with KIRC in cluster B had a higher probability of corresponding to genecluster C1, with a higher CUS and poorer prognosis. The different box plots also verify this conclusion (p <2.22e-16, **Figures 7D, E**). We created a correlation heatmap to explore the relationship between the CUS and immune cell infiltration (**Figure 7C**) and observed a negative but weak correlation between the CUS and most immune cells (p <0.05, except natural killer cells).

### CUS-related clinical subgroup analysis

To further explore the correlation between the CUS, prognosis, and clinical characteristics, we used box plots to show the differences in the CUS between different clinical characteristics and stacked histograms to show the proportion of each clinical characteristic in the high- and low-CUS groups. Patients who died had a higher CUS than those alive (p =2.4e-09, **Supplementary Figure 5A**); patients with histological grade G4 had a higher CUS than those with histological grade G1, G2 and G3 (p <0.1, **Supplementary Figure 5B**); patients with T4 had a higher CUS, while patients with T1 had a lower CUS (p <0.05, **Supplementary Figure 5C**); CUS did not show a significant difference in lymph node metastasis (**Supplementary Figure 5D**); patients with distant metastasis had a higher CUS than those without distant metastasis (p =0.009, **Supplementary Figure 5E**), and patients with clinical stage I had a lower CUS than those with clinical stage II, III and IV (p <0.05, **Supplementary Figure 5F**).

### Exploration of immunotherapy targets and efficacy based on CUS

**Figure 8:**
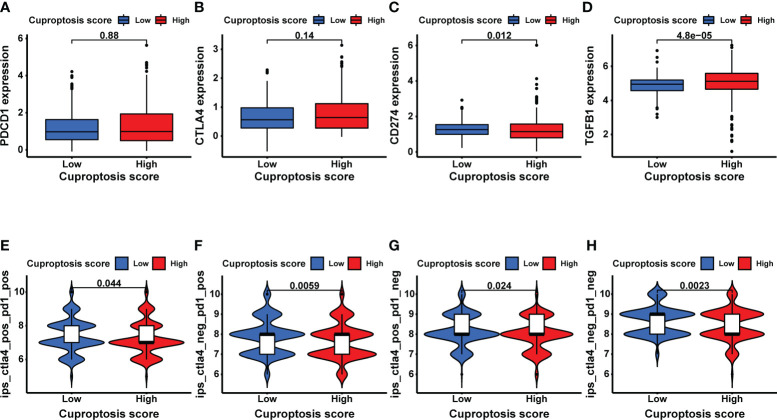
Exploratory analysis of immunotherapy based on CUS grouping. **(A–D)** The expression levels of immune target genes in different CUS groups: *PDCD1* **(A)**, *CTLA4* **(B)**, *CD274* **(C)** and *TGFB1* **(D)**. **(E–H)** The efficacy of 4 ICIs was predicted by IPS scores from the TCGA-KIRC cohort in TCIA database: ctla4_pos_pd1_pos **(E)**, ctla4_neg_pd1_pos **(F)**, ctla4_pos_pd1_neg **(G)** and ctla4_neg_pd1_neg **(H)**. CUS, cuproptosis score; ICI: immune checkpoint inhibitor; IPS, immune cell proportion score; pos, positive; neg, negative.

To explore the potential relationship between CUS and immunotherapy, we compared the expression levels of several common immune-related targets in the high- and low-CUS groups. In the high-CUS group, the *TGFB1* expression level was higher (p =4.8e-05, **Figure 8D**), whereas CD274 expression was lower (p =0.012, **Figure 8C**) than that in the low-CUS group. There were no significant differences in PDCD1 (**Figure 8A**) and CTLA4 (**Figure 8B**) expression levels between the high- and low-CUS groups. In addition, we downloaded the IPS of the TCGA-KIRC cohort from the TCIA database to explore differences in the efficacy of immunotherapy between the high- and low-CUS groups. The IPS of ctla4_pos_pd1_pos (**Figure 8E**), ctla4_neg_pd1_pos (**Figure 8F**), ctla4_pos_pd1_neg (**Figure 8G**), and ctla4_neg_pd1_neg (**Figure 8H**) was lower in the high-CUS group (p <0.05) than in the low-CUS group.

### Sensitivity analysis and efficacy prediction of targeted drugs

**Figure 9:**
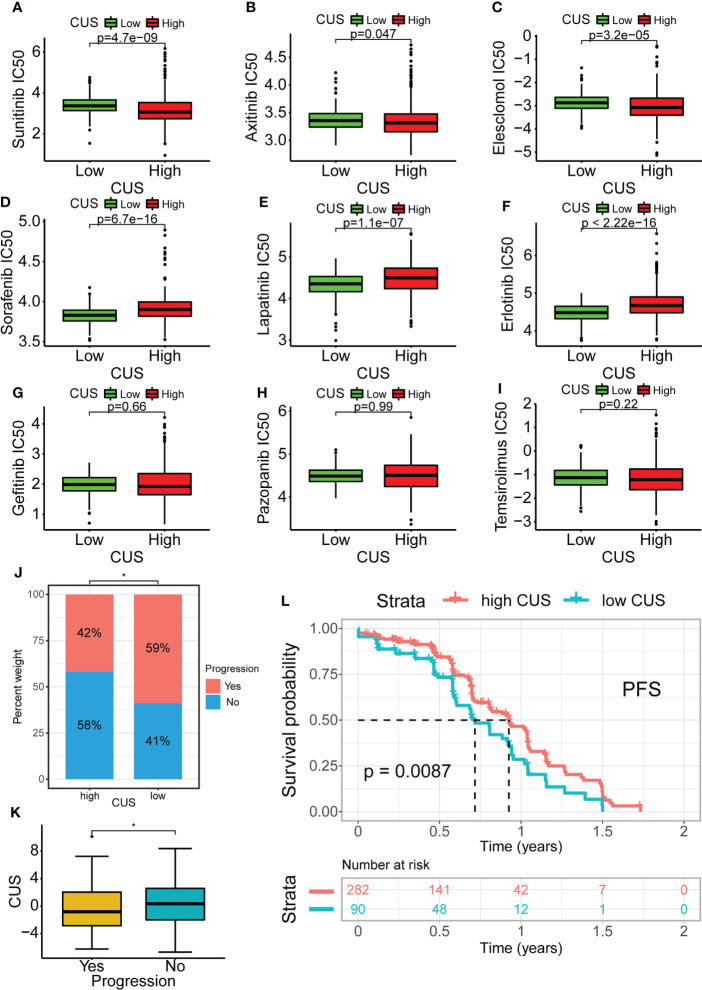
Drug susceptibility analysis of common targeted drugs and validation of targeted therapy cohort in KIRC. Based on cuproptosis grouping, drug sensitivity of Sunitinib**(A)**, Axitinib **(B)**, Elesclomol **(C)**, Sorafenib **(D)**, Lapatinib **(E)**, Erlotinib **(F)**, Gefitinib **(G)**, Pazopanib **(H)** and Temsirolimus **(I)** is conducted. **(J)** Obtaining data from the KIRC cohort using Sunitinib in NCT02684006. Proportional distribution of progression status in the high- and low-CUS groups. **(K)** Comparison of CUS levels between progression group and non-progression group. **(L)** Kaplan–Meier survival analysis between the high- and low-CUS groups (PFS, Log-rank test, p < 0.001). CUS, cuproptosis score; PFS: progression free survival; *p < 0.05.

To explore the impact of the CUS on targeted therapy, we first calculated the IC50 values of a variety of commonly used targeted drugs for advanced KIRC using the pRRophetic package and then predicted the sensitivity of targeted drugs. For sunitinib, axitinib, and elesclomol, the IC50 values of the high-CUS group were lower and showed higher sensitivity (p <0.05, **Figures 9D–F**). For sorafenib, erlotinib, and lapatinib, the high-CUS group had higher IC50 values and lower sensitivity (p <0.0001, **Figures 9A–C**). However, the IC50 values of gefitinib, pazopanib, and temsirolimus were not significantly different between the high- and low-CUS groups (**Figures 9G–I**).

Furthermore, we downloaded the cohort data from NCT02684006 using sunitinib-targeted therapy for prognostic analysis. The proportion of patients with progression in the high-CUS group was lower than that in the low-CUS group (42% vs. 59%, **Figure 9J**), and the patients who progressed had lower CUS than those who did not (p <0.05, **Figure 9K**). The results of the Kaplan-Meier survival analysis showed that the high-CUS group had a longer PFS than the low-CUS group (p =0.0087, **Figure 9L**).

Additionally, we systematically mined the targeted drugs associated with the seven CPGs using the GSCA database. Using the GDSC (**Supplementary Figure 6A**) and CTRP data sources (**Supplementary Figure 6B**), the top 30 targeted drugs whose sensitivity was most strongly correlated with the mRNA expression of the seven CPGs were determined.

### Identification and multi-level expression validation of potential CPGs

**Figure 10:**
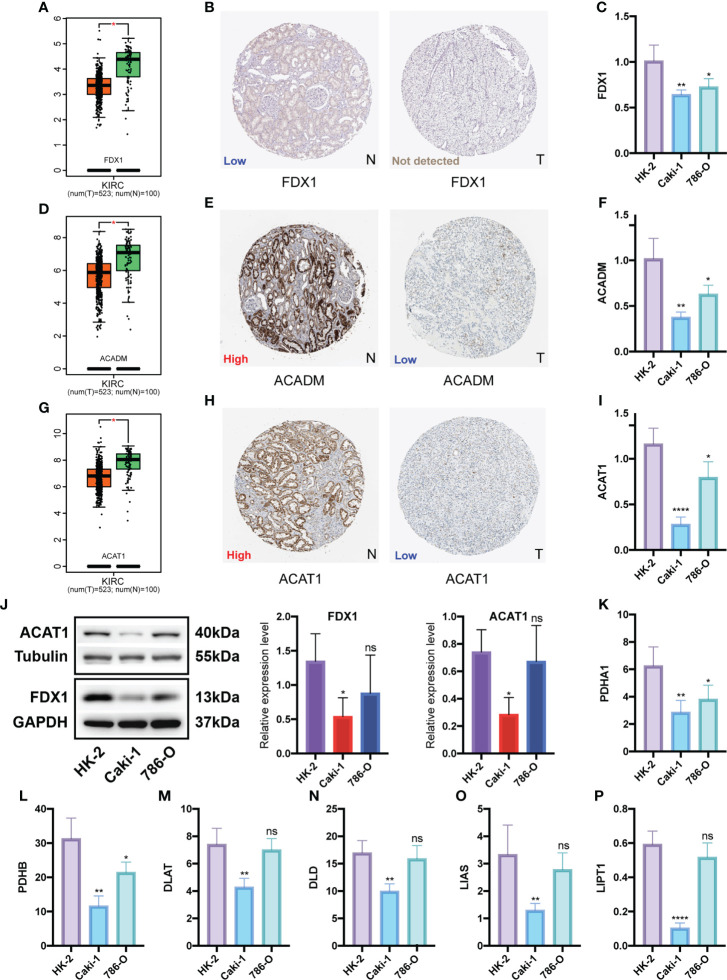
Differential expression validation of 7 CPGs and 2 potential CPGs between KIRC and normal samples. GEPIA database was used to compare the expression differences of *FDX1* **(A)**, *ACADM* **(D)** and *ACAT1* **(G)** between KIRC and normal kidney tissues. The HPA database showed the expression of *FDX1* **(B)**, *ACADM* **(E)** and *ACAT1* **(H)** at the tissue protein level by immunohistochemistry. Western Blot was used to compare the protein levels of *FDX1* and *ACAT1* in KIRC cells (Caki-1 and 786-0) and normal renal tubular epithelial cells (HK-2), and the results were semi-quantified by integrated density **(J)**. qRT-PCR was used to compare mRNA levels of *FDX1* **(C)**, *ACADM* **(F)**, *ACAT1* **(I)**, *PDHA1* **(K)**, *PDHB* **(L)**, *DLAT* **(M)**, *DLD* **(N)**, *LIAS* **(O)** and *LIPT1* **(P)** in KIRC cells (Caki-1, 786-0) and normal renal tubular epithelial cells (HK-2). CPG, cuproptosis-promoting gene; N, normal; T,tumor; ns, no significant difference, *p < 0.05, **p < 0.01, and ****p < 0.0001. Western Blot data are means ± SD, with n = 3; qRT-PCR data are means ± SD, with n = 4.

We identified two potential CPGs, *ACADM* and *ACAT1*, based on 31 CSRGs. We then performed differential expression validation of these two potential CPGs and seven identified CPGs (*FDX1*, *LIAS*, *LIPT1*, *DLD*, *DLAT*, *PDHA1* and *PDHB*). The GEPIA database showed that the expression of *FDX1* (**Figure 10A**), *ACADM* (**Figure 10D**) and *ACAT1* (**Figure 10G**) in KIRC was lower than that in the normal kidney tissue (p <0.05). The immunohistochemical results of *FDX1* (**Figure 10B**), *ACADM* (**Figure 10E**) and *ACAT1* (**Figure 10H**) in the HPA database showed that the protein expression of these genes significantly decreased in KIRC tissues compared to that in normal renal tubular epithelial tissues. In addition, we detected the expression differences of *FDX1* (**Figure 10C**), *ACADM* (**Figure 10F**), *ACAT1* (**Figure 10I**), *PDHA1* (**Figure 10K**), *PDHB* (**Figure 10L**), *DLAT* (**Figure 10M**), *DLD* (**Figure 10N**), *LIAS* (**Figure 10O**) and *LIPT1* (**Figure 10P**) between renal tubular epithelial cells (HK-2) and KIRC cells (Caki-1 and 786-O) using qRT-PCR. The expression of these genes was significantly down-regulated in Caki-1 cells compared to that in HK-2 cells (p <0.05). Finally, western blot results indicated that *FDX1* and *ACAT1* expression was higher in HK-2 cells than in Caki-1 cells, but there was no significant difference between 786-O and HK-2 cells (**Figure 10J**).

### Proliferation functional validation of *FDX1* and validation of cuproptosis in KIRC cells

**Figure 11:**
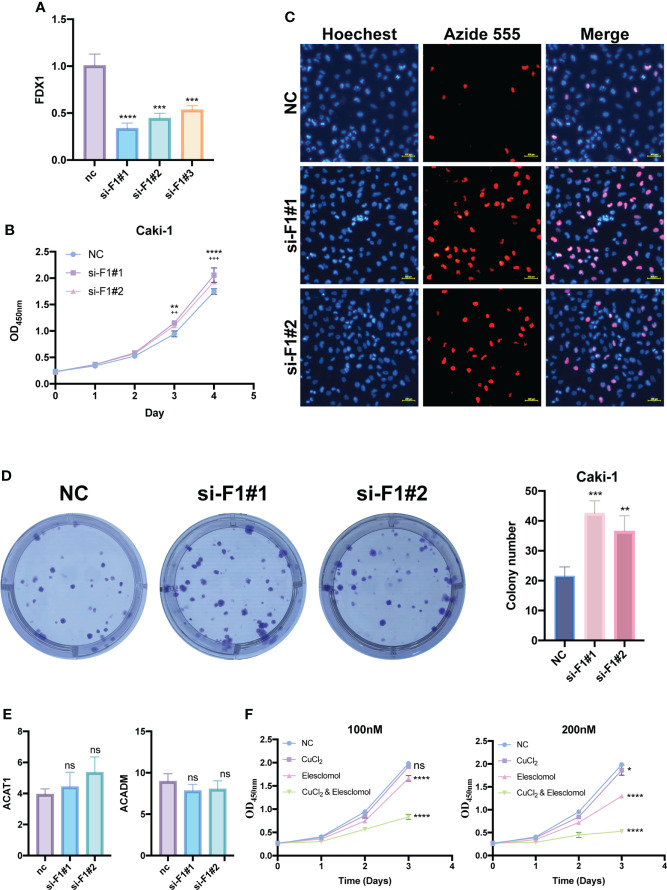
Proliferation functional validation of *FDX1* and validation of cuproptosis in KIRC cells. **(A)** Silencing efficiency of *FDX1* by qRT-PCR. CCK8 assay **(B)**, EDU assay **(C)** and Colony formation assay **(D)** show the effect of *FDX1* knockdown on the proliferation of Caki-1 cells. * is for si-F1#1, ^+^ is for si-F1#2. **(E)** Changes in *ACADM* and *ACAT1* mRNA expression levels after knockdown of *FDX1* by qRT-PCR. **(F)** The effects of elesclomol and CuCl_2_ on Caki-1 cell death were explored by CCK8 assay. si-F1#1, si-FDX1#1; si-F1#2, si-FDX1#2; ns, no significant difference, *p < 0.05, **p < 0.01, ***p < 0.001 and ****p < 0.0001; ^++^p < 0.01 and ^+++^ p < 0.001; CCK8 assay and Colony formation assay data are means ± SD, with n = 3; qRT-PCR data are means ± SD, with n = 4.

To further investigate the function of *FDX1*, a key cuproptosis gene, in KIRC, we silenced *FDX1* to detect its effect on Caki-1 cells’ proliferation. Subsequently, si-FDX1#1 and si-FDX1#2 were determined for further study because they showed higher silencing efficacy compared to NC-transfected cells (**Figure 11A**). CCK8 and EdU assays showed that FDX1 depletion promoted KIRC cell growth (**Figures 11B, C**), and the clone formation assay demonstrated that FDX1 improves the proliferative capacity of KIRC cells (**Figure 11D**). To figure out the relationship between FDX1 and two potential CPGs, we detected the expression of ACADM and ACAT1 in transfected Caki-1 cells. However, no significant expression difference was found (**Figure 11E**).

Elesclomol has recently been found to be a potent copper ionophore. We performed the CCK8 assay of elesclomol and CuCl_2_ to test whether there is cuproptosis in Caki-1 cells. Compared with the negative control group, the CuCl_2_ group showed slight cytotoxicity, but was not very significant, the elesclomol group showed certain cytotoxicity, while CuCl_2_& elesclomol group had a significantly enhanced cytotoxicity on Caki-1 cells (**Figure 11F**).

## Discussion

KIRC is insensitive to both radiotherapy and chemotherapy (5), and targeted therapy has been the mainstay of treatment of advanced KIRC. In recent years, the development of ICIs has changed the treatment landscape of advanced KIRC and ICIs have become the first-line treatment option (31). Single-agent pembrolizumab has shown antitumor activity in the first-line treatment of patients with advanced KIRC according to the phase II KEYNOTE-427 study (32). In the phase III CheckMate 214 clinical trial, nivolumab combined with ipilimumab improved OS in patients with intermediate- or low-risk previously untreated advanced KIRC (33). Targeted therapy combined with immunotherapy has become the first-line treatment for metastatic or unresectable KIRC and tends to gradually replace targeted therapy alone (9). However, there is still a lack of effective targets to help patients with metastatic or unresectable KIRC more precisely and to individually select therapeutic drugs.

Cuproptosis is an emerging cell death mechanism, which is closely related to mitochondrial metabolism and the TCA cycle, which is different from other death modes such as ferroptosis, necroptosis, and pyroptosis. It is precise because of the unique metabolic characteristics of cuproptosis that KIRC is considered to be more closely related to cuproptosis than to several other cell death modes. These previously discovered cell death modes have been found to have many predictive models and molecular typing constructed in KIRC (34–36), and cuproptosis still has great research prospects for KIRC typing. *FDX1*, a key gene that promotes cuproptosis, functions as an upstream regulator of protein lipoylation. The lipoylated component of the TCA cycle directly binds to Cu ions to activate cuproptosis (13). Therefore, both Cu ions and mitochondrial respiration are key factors in cuproptosis. KIRC has a unique metabolic profile that exhibits the classic Warburg effect *in vivo* (37). This means that KIRC exhibits marked inhibition of glucose oxidation and activation of aerobic glycolysis (38). Based on the correlation between worsening prognosis and metabolic shifts in patients with KIRC, including decreased TCA cycle activity, increased dependence on pentose phosphate shunt, decreased AMP-activated protein kinase, increased glutamine transport, and fatty acid production (39), we hypothesized that cuproptosis may be suppressed in KIRC. Differential analysis verified our conjecture, and seven CPGs displayed varying degrees of low expression in KIRC. This result suggests an association between cuproptosis and the gene map of KIRC.

First, we selected seven CPGs with similar functions and strong correlations and identified two clusters with different biological and clinical characteristics in the comprehensive cohort of TCGA-KIRC and GSE29609. Cluster B showed a poorer prognosis and a higher level of immune infiltration than cluster A, which includes immune-promoting cells, such as CD4+ T cells, CD8+ T cells, and immune-suppressing cells, such as MDSCs and macrophages. Next, we performed a differential analysis of the two subtypes and identified 31 DEGs. Enrichment analysis revealed that DEGs were associated with various metabolic pathways, including the PPAR signaling pathway, carbon metabolism, TCA cycle, fatty acid degradation, and the PI3K-Akt signaling pathway. The PPAR signaling pathway is related to mitochondrial metabolism, fatty acylation of proteins involves the attachment of fatty acids to peptide chains in the form of fatty acyl groups, and lipoylation of key proteins in the mitochondrial TCA cycle is the core mechanism of cuproptosis. The downstream target of PI3K/Akt is a mammalian target of rapamycin (mTOR), which is key to the treatment of advanced KIRC. This finding also provided us with more potential molecular mechanisms of cuproptosis in KIRC.

Using univariate Cox regression analysis, we selected all 31 DEGs for CSRGs. We further constructed two new clusters based on the 31 CSRGs. Among them, geneclusterC1 had the poorest prognosis. To quantify the status of cuproptosis, predict the prognosis of KIRC more accurately, and guide clinical decision-making, we constructed the CUS using the Boruta algorithm combined with PCA. The patients in the high-CUS group had a poorer prognosis. The expression of immune-related targets showed that patients in the high-CUS group had significantly higher expression of *TGFB1*, which is one of the ligands of the TGFB pathway and is the most prevalent isoform expressed in many human tumors. Regulatory T cells (Tregs) exert a contact-dependent inhibitory effect on immune cells by producing TGFB1. On the surface of Tregs, TGFB1 binds to the membrane protein GARP in its inactive form. Monoclonal antibodies against the GARP/TGFB1 complex alone or in combination with antibodies targeting the CTLA4 or PD1/PD-L1 pathways can improve the efficacy of immunotherapy (40). In addition, selective inhibition of *TGFB1* may alter resistance to anti-PD-1 therapy by altering the tumor immune environment (41). Galunisertib is a novel inhibitor of TGF-β receptor 1. Phase 1 studies have demonstrated the safety of galunisertib and its antitumor activity in patients with glioma (42). In addition, combination therapy with *TGFB* and ICIs is in the clinical drug development stage for hepatocellular carcinoma, non-small cell lung cancer, and pancreatic cancer (NCT02423343; NCT02734160) (43). We then used TCIA data to predict the efficacy of immunotherapy and observed that the patients in the high-CUS group showed poorer results for all four ICIs. This finding suggests that the effect of different CUS values on the efficacy of immunotherapy is not dependent on *PD1* and *CTLA4*, but may be dependent on *TGFB1*. This finding is consistent with the results of our study. Therefore, our findings suggest that the combination of *TGFB1* inhibitors and immunotherapy may improve the efficacy of immunotherapy for patients in the high-CUS group.

Next, we explored targeted therapy. The results of the IC50 drug susceptibility analysis suggested that the patients in the high-CUS group were more likely to develop resistance to sorafenib, erlotinib, and lapatinib, but they were more sensitive to sunitinib and axitinib. Thus, although patients in the high-CUS group have poorer prognosis and immunotherapy efficacy, sunitinib and axitinib may bring clinical benefits. Sunitinib monotherapy is the classic first-line therapy for advanced KIRC (7, 44). We further performed prognostic validation in a cohort of patients with sunitinib-treated advanced KIRC in NCT02684006. After sunitinib treatment, the high-CUS group showed slower progression and even reversed the original poor prognosis in the PFS curve. This suggests that sunitinib may be one of the few treatment options for patients with high CUS.

Drug susceptibility analysis also revealed that the patients in the high-CUS group were more sensitive to elesclomol. Cancer cells with high mitochondrial respiration are more prone to cuproptosis, and copper ionophores represented by elesclomol may present a new cancer therapy (13). Current clinical trials of elesclomol have not achieved satisfactory results (14, 45), however, we expect to discover new ways to activate cuproptosis. The distinct metabolic profile of KIRC suggests that activation of cuproptosis may require a combination of elesclomol and aerobic glycolysis inhibitors. Studies have shown a relationship between metabolic and immune activity in KIRC (46), whereas *TGFB* was found to promote aerobic glycolysis in renal fibroblasts (47). *TGFB1* may play a role in this relation; therefore, combination therapy may benefit from *TGFB1* inhibitors.

Finally, we identified two potential CPGs from 31 CSRGs: *ACADM* and *ACAT1*. We verified that these two genes were weakly expressed in KIRC compared to the normal samples through three levels of validation in the GEPIA database, HPA database, and qRT-PCR results. The main function of *ACADM* is to catalyze the initial step of the mitochondrial fatty acid β-oxidation pathway. Inhibition of *ACADM* can promote dysregulation of fatty acid oxidation, leading to hepatocellular carcinoma progression (48). *ACAT1* is a key enzyme that catalyzes the production of mitochondrial ketone bodies. Combination therapy with an *ACAT1* inhibitor and anti-PD-1 antibody showed better efficacy than immunotherapy alone in controlling tumor progression (49). Previous studies show that *ACADM* and *ACAT1* are protective tumor suppressors of KIRC, which is consistent with our findings (50, 51).

In summary, we performed cluster analysis for typing based on CPGs and constructed a score that quantifies the cuproptosis status. The CUS can effectively predict the prognosis and efficacy of targeted therapy and immunotherapy. In conjunction with the 2021 National Comprehensive Cancer Network (NCCN) guidelines (52), we developed a detailed treatment strategy for treatment options for patients with metastatic or unresectable KIRC. For patients in the low-CUS group, conventional targeted therapy combined with immunotherapy strategies recommended by the guidelines can be used as the first-line treatment. Note that among the targeted therapy drugs, axitinib and sunitinib can be avoided if possible. For the first-line treatment of patients in the high-CUS group, we recommend a triple-drug combination of axitinib, pembrolizumab, and a *TGFB1* inhibitor. Sunitinib monotherapy is also a feasible treatment option. Axitinib monotherapy can be used as a second-line therapy.

To further explore the relationship between the cuproptosis gene and KIRC, we first verified the differential expression of the CPGs at mRNA and protein levels. Next, we silenced *FDX1* in Caki-1 cells and found that the proliferation of Caki-1 cells was significantly promoted. Finally, to provide preliminary evidence that Cuproptosis may be present in KIRC, we designed CCK8 assays with reference to the research of Tsvetkov P et al. (53). We found that when copper was added to elesclomol at a molar ratio of 1:1, it significantly reduced the activity of KIRC cells. These results suggest that the delivery of large amounts of copper ions into cells by elesclomol may trigger cuproptosis in KIRC.

The study has some limitations. First, more cohorts of immunotherapy and targeted therapy are needed to validate and optimize the conclusions and improve the predictive power of the scoring system. Second, *TGFB1* inhibitors, elesclomol, and aerobic glycolysis inhibitors are new therapeutic agents based on cuproptosis. Further basic clinical trials are required to explore the efficacy of these agents.

## Conclusion

In this study, a scoring system for cuproptosis—CUS was constructed, which developed a novel and precise strategy for the selection of targeted therapy and immunotherapy in patients with advanced KIRC, and also provided new insights into the relationship among cuproptosis, metabolism and immunity in KIRC.

## Data availability statement

The datasets presented in this study can be found in online repositories. The names of the repository/repositories and accession number(s) can be found in the article/**Supplementary Material**. Due to the large size of the original data and code files, the complete original data and code can be downloaded from https://www.jianguoyun.com/p/DVP0rksQ3rToChjO1M4EIAA. If the link does not work, please contact our corresponding author's email address and we will provide it.

## Author contributions

KC designed the study, GZ and XC conducted the data analysis, and wrote the manuscript. GZ, XC, and JF participated in and contributed to the experiments of this study. JF, PT, and AC participated in manuscript revision. All authors read and approved the final manuscript.

## Funding

This work was supported by the National Natural Science Foundation of China (81874137), Funds for International Cooperation and Exchange of the National Natural Science Foundation of China (GZ1699), key research and development projects in Hunan Province (2022SK2022), the science and technology innovation Program of Hunan Province (2020RC4011), the Hunan Province Science and Technology Talent Promotion Project (2019TJ-Q10), Scientific research project of Hunan Provincial Health Commission (202209034683), Young Scholars of “Furong Scholar Program” in Hunan Province, and the Wisdom Accumulation and Talent Cultivation Project of the Third Xiangya Hospital of Central South University (BJ202001).

## Acknowledgments

We sincerely thank the public databases, including GEPIA2, GSCA, HPA, UCSC Xena and GEO for providing open access.

## Conflict of interest

The authors declare that the research was conducted in the absence of any commercial or financial relationships that could be construed as a potential conflict of interest.

## Publisher’s note

All claims expressed in this article are solely those of the authors and do not necessarily represent those of their affiliated organizations, or those of the publisher, the editors and the reviewers. Any product that may be evaluated in this article, or claim that may be made by its manufacturer, is not guaranteed or endorsed by the publisher.

## Supplementary material

Supplementary Figure 1 Genetic, transcriptional and post-transcriptional alterations of CPGs in KIRC in GSCA database. **(A)** Expression level of seven CPGs. **(B)** Frequencies of SNV among CPGs. **(C)** Percentage of various types of CNV in CPGs. **(D)** Spearman correlation between mRNA expression and CNV levels of CPGs. **(E)** Spearman correlation between mRNA expression and methylation levels of CPGs. **(F)** Differences in methylation levels of CPGs. CPG: cuproptosis-promoting gene.

Supplementary Figure 2 Detailed results of consensus clustering. **(A–C)** Detailed Results of consensus clustering to construct CPGs subtypes: cumulative distribution curve **(A)**, area under the cumulative distribution curve **(B)** and tracking plot **(C)**. **(D–F)** Detailed Results of consensus clustering to construct CSRGs gene subtypes: cumulative distribution curve **(D)**, area under the cumulative distribution curve **(E)** and tracking plot **(F)**. CPG, cuproptosis-promoting gene; CSRG, cuproptosis subtypes related gene.

Supplementary Figure 3 Difference comparison of enriched pathways of two different CPG subtypes by GSVA. **(A–C)** Heatmaps comparing GSVA pathway scores for two CPG subtypes from three items: HALLMARK **(A)**, KEGG **(B)**, and Reactome **(C)**. CPG, cuproptosis-promoting gene.

Supplementary Figure 4 Differences in clinicopathological and biological features between two gene subtypes. **(A)** Expression differences of 31 CSRGs between the two gene subtypes. **(B)** Heatmap of the distribution of clinicopathological features and CSRG expression between two different gene subtypes. CSRG, cuproptosis subtypes related gene; ***p < 0.001.

Supplementary Figure 5 Clinical subgroup analysis of CUS in KIRC. **(A–G)** Finding the relationship between six clinical features and CUS by difference comparison and ratio distribution: fustat **(A)**, Grade **(B)**, T_stage **(C)**, N_stage **(D)**, M_stage **(E)** and Stage **(F)**. CUS, cuproptosis score.

Supplementary Figure 6 Observation of the sensitivity of CPGs to targeted drugs using the GSCA database. **(A)** Correlation between GDSC drug sensitivity and CPGs mRNA expression. **(B)** Correlation between CTRP drug sensitivity and CPGs mRNA expression. CPG, cuproptosis-promoting gene.

## Abbreviations

CNV, copy number variation; CPG, cuproptosis-promoting gene; CSRG, cuproptosis subtypes related gene; CUS, cuproptosis score; Cu, Copper; CCK8, Cell Counting Kit-8; DEG, differentially expressed gene; EdU, Ethynyl-2’-deoxyuridine; GEO, Gene Expression Omnibus; GEPIA, Gene Expression Profiling Interactive Analysis; GSCA, Gene Set Cancer Analysis; HPA, Human Protein Atlas; IC50, half maximal inhibitory concentration; ICI, immune checkpoint inhibitor; IPS, Immunophenoscore; KEGG, Kyoto Encyclopedia of Genes and Genomes; KIRC, kidney renal clear cell carcinoma; OS, overall survival; PCA, principal component analysis; qRT-PCR, quantitative reverse transcription polymerase chain reaction; SNV, single nucleotide variation; ssGSEA, single sample gene set enrichment analysis; TCA, tricarboxylic acid cycle; TCGA, The Cancer Genome Atlas; TCIA, The Cancer Immunome Atlas.

## References

[1] Linehan WM Ricketts CJ. The cancer genome atlas of renal cell carcinoma: findings and clinical implications. Nat Rev Urol (2019) 16(9):539–52. doi: 10.1038/s41585-019-0211-5 31278395

[2] Rini BI Campbell SC Escudier B. Renal cell carcinoma. Lancet (2009) 373(9669):1119–32. doi: 10.1016/s0140-6736(09)60229-4 19269025

[3] Fisher R Gore M Larkin J. Current and future systemic treatments for renal cell carcinoma. Semin Cancer Biol (2013) 23(1):38–45. doi: 10.1016/j.semcancer.2012.06.004 22705280

[4] Master VA Gottschalk AR Kane C Carroll PR. Management of isolated renal fossa recurrence following radical nephrectomy. J Urol (2005) 174(2):473–7. doi: 10.1097/01.ju.0000165574.62188.d0 16006867

[5] Jacobsohn KM Wood CG. Adjuvant therapy for renal cell carcinoma. Semin Oncol (2006) 33(5):576–82. doi: 10.1053/j.seminoncol.2006.06.005 17045086

[6] Bagcchi S. Sunitinib still fi rst-line therapy for metastatic renal cancer. Lancet Oncol (2014) 15(10):e420. doi: 10.1016/s1470-2045(14)70366-3 25328943

[7] Ravaud A Motzer RJ Pandha HS George DJ Pantuck AJ Patel A. Adjuvant sunitinib in high-risk renal-cell carcinoma after nephrectomy. N Engl J Med (2016) 375(23):2246–54. doi: 10.1056/NEJMoa1611406 27718781

[8] Hsieh JJ Purdue MP Signoretti S Swanton C Albiges L Schmidinger M. Renal cell carcinoma. Nat Rev Dis Primers (2017) 3:17009. doi: 10.1038/nrdp.2017.9 28276433 PMC5936048

[9] Rini BI Plimack ER Stus V Gafanov R Hawkins R Nosov D. Pembrolizumab plus axitinib versus sunitinib for advanced renal-cell carcinoma. N Engl J Med (2019) 380(12):1116–27. doi: 10.1056/NEJMoa1816714 30779529

[10] Yu EM Linville L Rosenthal M Aragon-Ching JB. A contemporary review of immune checkpoint inhibitors in advanced clear cell renal cell carcinoma. Vaccines (Basel) (2021) 9(8):919. doi: 10.3390/vaccines9080919 34452045 PMC8402652

[11] Tsang T Davis CI Brady DC. Copper biology. Curr Biol (2021) 31(9):R421–r427. doi: 10.1016/j.cub.2021.03.054 33974864

[12] Saporito-Magriñá CM Musacco-Sebio RN Andrieux G Kook L Orrego MT Tuttolomondo MV. Copper-induced cell death and the protective role of glutathione: the implication of impaired protein folding rather than oxidative stress. Metallomics (2018) 10(12):1743–54. doi: 10.1039/c8mt00182k 30311620

[13] Tsvetkov P Coy S Petrova B Dreishpoon M Verma A Abdusamad M. Copper induces cell death by targeting lipoylated TCA cycle proteins. Science (2022) 375(6586):1254–61. doi: 10.1126/science.abf0529 PMC9273333 35298263

[14] O'Day SJ Eggermont AM Chiarion-Sileni V Kefford R Grob JJ Mortier L. Final results of phase III SYMMETRY study: randomized, double-blind trial of elesclomol plus paclitaxel versus paclitaxel alone as treatment for chemotherapy-naive patients with advanced melanoma. J Clin Oncol (2013) 31(9):1211–8. doi: 10.1200/jco.2012.44.5585 23401447

[15] Teng R Liu Z Tang H Zhang W Chen Y Xu R. HSP60 silencing promotes warburg-like phenotypes and switches the mitochondrial function from ATP production to biosynthesis in ccRCC cells. Redox Biol (2019), 24:101218. doi: 10.1016/j.redox.2019.101218 31112866 PMC6526248

[16] Voli F Valli E Lerra L Kimpton K Saletta F Giorgi FM. Intratumoral copper modulates PD-L1 expression and influences tumor immune evasion. Cancer Res (2020) 80(19):4129–44. doi: 10.1158/0008-5472.Can-20-0471 32816860

[17] Liu CJ Hu FF Xia MX Han L Zhang Q Guo AY. GSCALite: a web server for gene set cancer analysis. Bioinformatics (2018) 34(21):3771–2. doi: 10.1093/bioinformatics/bty411 29790900

[18] Johnson WE Li C Rabinovic A. Adjusting batch effects in microarray expression data using empirical bayes methods. Biostatistics (2007) 8(1):118–27. doi: 10.1093/biostatistics/kxj037 16632515

[19] Wilkerson MD Hayes DN. ConsensusClusterPlus: a class discovery tool with confidence assessments and item tracking. Bioinformatics (2010) 26(12):1572–3. doi: 10.1093/bioinformatics/btq170 PMC2881355 20427518

[20] Hänzelmann S Castelo R Guinney J. GSVA: gene set variation analysis for microarray and RNA-seq data. BMC Bioinf (2013) 14:7. doi: 10.1186/1471-2105-14-7 PMC3618321 23323831

[21] Yoshihara K Shahmoradgoli M Martínez E Vegesna R Kim H Torres-Garcia W. Inferring tumour purity and stromal and immune cell admixture from expression data. Nat Commun (2013), 4:2612. doi: 10.1038/ncomms3612 24113773 PMC3826632

[22] Subramanian A Tamayo P Mootha VK Mukherjee S Ebert BL Gillette MA. Gene set enrichment analysis: a knowledge-based approach for interpreting genome-wide expression profiles. Proc Natl Acad Sci U.S.A. (2005) 102(43):15545–50. doi: 10.1073/pnas.0506580102 PMC1239896 16199517

[23] Ritchie ME Phipson B Wu D Hu Y Law CW Shi W. Limma powers differential expression analyses for RNA-sequencing and microarray studies. Nucleic Acids Res (2015) 43(7):e47. doi: 10.1093/nar/gkv007 25605792 PMC4402510

[24] Yu G Wang LG Han Y He QY. clusterProfiler: an r package for comparing biological themes among gene clusters. Omics (2012) 16(5):284–7. doi: 10.1089/omi.2011.0118 PMC3339379 22455463

[25] Degenhardt F Seifert S Szymczak S. Evaluation of variable selection methods for random forests and omics data sets. Brief Bioinform (2019) 20(2):492–503. doi: 10.1093/bib/bbx124 29045534 PMC6433899

[26] Charoentong P Finotello F Angelova M Mayer C Efremova M Rieder D. Pan-cancer immunogenomic analyses reveal genotype-immunophenotype relationships and predictors of response to checkpoint blockade. Cell Rep (2017) 18(1):248–62. doi: 10.1016/j.celrep.2016.12.019 28052254

[27] Geeleher P Cox N Huang RS. pRRophetic: an r package for prediction of clinical chemotherapeutic response from tumor gene expression levels. PloS One (2014) 9(9):e107468. doi: 10.1371/journal.pone.0107468 25229481 PMC4167990

[28] Motzer RJ Robbins PB Powles T Albiges L Haanen JB Larkin J. Avelumab plus axitinib versus sunitinib in advanced renal cell carcinoma: biomarker analysis of the phase 3 JAVELIN renal 101 trial. Nat Med (2020) 26(11):1733–41. doi: 10.1038/s41591-020-1044-8 PMC8493486 32895571

[29] GTEx Consortium. The genotype-tissue expression (GTEx) project. Nat Genet (2013) 45(6):580–5. doi: 10.1038/ng.2653 PMC4010069 23715323

[30] Tang Z Kang B Li C Chen T Zhang Z. GEPIA2: an enhanced web server for large-scale expression profiling and interactive analysis. Nucleic Acids Res (2019) 47(W1):W556–w560. doi: 10.1093/nar/gkz430 31114875 PMC6602440

[31] Lin E Liu X Liu Y Zhang Z Xie L Tian K. Roles of the dynamic tumor immune microenvironment in the individualized treatment of advanced clear cell renal cell carcinoma. Front Immunol (2021) 12:653358. doi: 10.3389/fimmu.2021.653358 33746989 PMC7970116

[32] McDermott DF Lee JL Bjarnason GA Larkin JMG Gafanov RA Kochenderfer MD. Open-label, single-arm phase II study of pembrolizumab monotherapy as first-line therapy in patients with advanced clear cell renal cell carcinoma. J Clin Oncol (2021) 39(9):1020–8. doi: 10.1200/jco.20.02363 PMC8078336 33529051

[33] Cella D Grünwald V Escudier B Hammers HJ George S Nathan P. Patient-reported outcomes of patients with advanced renal cell carcinoma treated with nivolumab plus ipilimumab versus sunitinib (CheckMate 214): a randomised, phase 3 trial. Lancet Oncol (2019) 20(2):297–310. doi: 10.1016/s1470-2045(18)30778-2 30658932 PMC6701190

[34] Xing XL Liu Y Liu J Zhou H Zhang H Zuo Q. Comprehensive analysis of ferroptosis- and immune-related signatures to improve the prognosis and diagnosis of kidney renal clear cell carcinoma. Front Immunol (2022) 13:851312. doi: 10.3389/fimmu.2022.851312 35619698 PMC9128788

[35] Xin S Mao J Duan C Wang J Lu Y Yang J. Identification and quantification of necroptosis landscape on therapy and prognosis in kidney renal clear cell carcinoma. Front Genet (2022) 13:832046. doi: 10.3389/fgene.2022.832046 35237304 PMC8882778

[36] Sun Z Jing C Guo X Zhang M Kong F Wang Z. Comprehensive analysis of the immune infiltrates of pyroptosis in kidney renal clear cell carcinoma. Front Oncol (2021), 11:716854. doi: 10.3389/fonc.2021.716854 34568046 PMC8459616

[37] Linehan WM Srinivasan R Schmidt LS. The genetic basis of kidney cancer: a metabolic disease. Nat Rev Urol (2010) 7(5):277–85. doi: 10.1038/nrurol.2010.47 PMC2929006 20448661

[38] Courtney KD Bezwada D Mashimo T Pichumani K Vemireddy V Funk AM. Isotope tracing of human clear cell renal cell carcinomas demonstrates suppressed glucose oxidation in vivo. Cell Metab (2018) 28(5):793–800.e2. doi: 10.1016/j.cmet.2018.07.020 30146487 PMC6221993

[39] Cancer Genome Atlas Research Network. Comprehensive molecular characterization of clear cell renal cell carcinoma. Nature (2013) 499(7456):43–9. doi: 10.1038/nature12222 PMC3771322 23792563

[40] Cuende J Liénart S Dedobbeleer O van der Woning B De Boeck G Stockis J. Monoclonal antibodies against GARP/TGF-β1 complexes inhibit the immunosuppressive activity of human regulatory T cells in vivo. Sci Transl Med (2015) 7(284):284ra56. doi: 10.1126/scitranslmed.aaa1983 25904740

[41] Martin CJ Datta A Littlefield C Kalra A Chapron C Wawersik S. Selective inhibition of TGFβ1 activation overcomes primary resistance to checkpoint blockade therapy by altering tumor immune landscape. Sci Transl Med (2020) 12(536): eaay8456. doi: 10.1126/scitranslmed.aay8456 32213632

[42] Rodon J Carducci MA Sepulveda-Sánchez JM Azaro A Calvo E Seoane J. First-in-human dose study of the novel transforming growth factor-β receptor I kinase inhibitor LY2157299 monohydrate in patients with advanced cancer and glioma. Clin Cancer Res (2015) 21(3):553–60. doi: 10.1158/1078-0432.Ccr-14-1380 PMC4337847 25424852

[43] Holmgaard RB Schaer DA Li Y Castaneda SP Murphy MY Xu X. Targeting the TGFβ pathway with galunisertib, a TGFβRI small molecule inhibitor, promotes anti-tumor immunity leading to durable, complete responses, as monotherapy and in combination with checkpoint blockade. J Immunother Cancer (2018) 6(1):47. doi: 10.1186/s40425-018-0356-4 29866156 PMC5987416

[44] Staehler M Motzer RJ George DJ Pandha HS Donskov F Escudier B. Adjuvant sunitinib in patients with high-risk renal cell carcinoma: safety, therapy management, and patient-reported outcomes in the s-TRAC trial. Ann Oncol (2018) 29(10):2098–104. doi: 10.1093/annonc/mdy329 PMC6247664 30412222

[45] Monk BJ Kauderer JT Moxley KM Bonebrake AJ Dewdney SB Secord AA. A phase II evaluation of elesclomol sodium and weekly paclitaxel in the treatment of recurrent or persistent platinum-resistant ovarian, fallopian tube or primary peritoneal cancer: An NRG oncology/gynecologic oncology group study. Gynecol Oncol (2018) 151(3):422–7. doi: 10.1016/j.ygyno.2018.10.001 PMC6392076 30309721

[46] Wang Y Zheng XD Zhu GQ Li N Zhou CW Yang C. Crosstalk between metabolism and immune activity reveals four subtypes with therapeutic implications in clear cell renal cell carcinoma. Front Immunol (2022) 13:861328. doi: 10.3389/fimmu.2022.861328 35479084 PMC9035905

[47] Ding H Jiang L Xu J Bai F Zhou Y Yuan Q. Inhibiting aerobic glycolysis suppresses renal interstitial fibroblast activation and renal fibrosis. Am J Physiol Renal Physiol (2017) 313(3):F561–f575. doi: 10.1152/ajprenal.00036.2017 28228400

[48] Ma APY Yeung CLS Tey SK Mao X Wong SWK Ng TH. Suppression of ACADM-mediated fatty acid oxidation promotes hepatocellular carcinoma *via* aberrant CAV1/SREBP1 signaling. Cancer Res (2021) 81(13):3679–92. doi: 10.1158/0008-5472.Can-20-3944 33975883

[49] Yang W Bai Y Xiong Y Zhang J Chen S Zheng X. Potentiating the antitumour response of CD8(+) T cells by modulating cholesterol metabolism. Nature (2016) 531(7596):651–5. doi: 10.1038/nature17412 PMC4851431 26982734

[50] Xiao H Chen P Zeng G Xu D Wang X Zhang X. Three novel hub genes and their clinical significance in clear cell renal cell carcinoma. J Cancer (2019) 10(27):6779–91. doi: 10.7150/jca.35223 PMC6909945 31839812

[51] Chen L Peng T Luo Y Zhou F Wang G Qian K. ACAT1 and metabolism-related pathways are essential for the progression of clear cell renal cell carcinoma (ccRCC), as determined by Co-expression network analysis. Front Oncol (2019) 9:957. doi: 10.3389/fonc.2019.00957 31649873 PMC6795108

[52] Motzer RJ Jonasch E Boyle S Carlo MI Manley B Agarwal N. NCCN guidelines insights: Kidney cancer, version 1.2021. J Natl Compr Canc Netw (2020) 18(9):1160–70. doi: 10.6004/jnccn.2020.0043 PMC10191771 32886895

[53] Tsvetkov P Detappe A Cai K Keys HR Brune Z Ying W. Mitochondrial metabolism promotes adaptation to proteotoxic stress. Nat Chem Biol (2019) 15(7):681–9. doi: 10.1038/s41589-019-0291-9 PMC8183600 31133756

