# Supplementary figures and images for: Cuproptosis status affects treatment options about immunotherapy and targeted therapy for patients with kidney renal clear cell carcinoma
Source: Front Immunol. 2022 Aug 19;13:954440. doi: 10.3389/fimmu.2022.954440 (PMC9437301; doi:10.3389/fimmu.2022.954440)

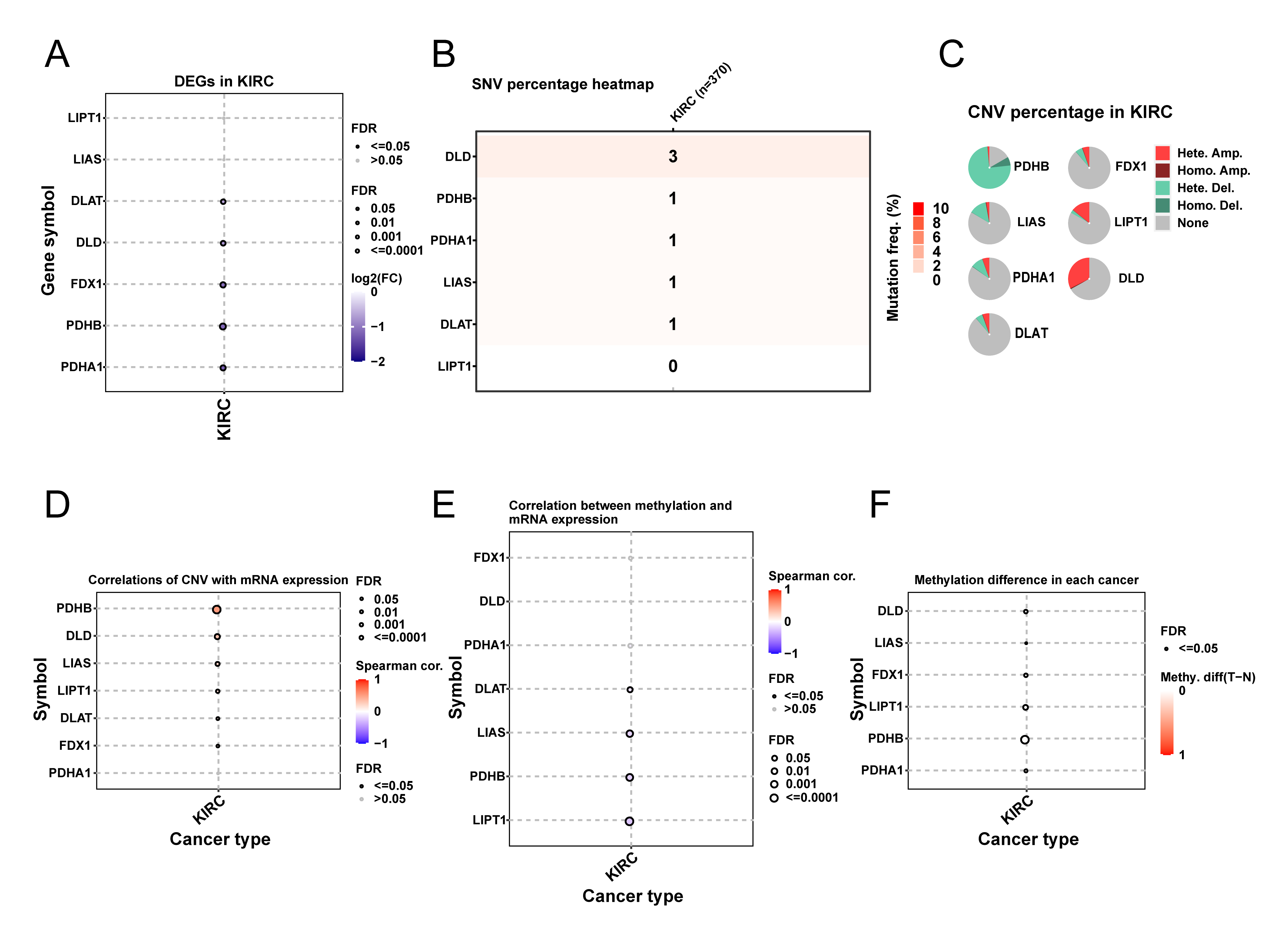

Supplement: Supplementary Figure 1 — Genetic, transcriptional and post-transcriptional alterations of CPGs in KIRC in GSCA database. (A) Expression level of seven CPGs. (B) Frequencies of SNV among CPGs. (C) Percentage of various types of CNV in CPGs. (D) Spearman correlation between mRNA expression and CNV levels of CPGs. (E) Spearman correlation between mRNA expression and methylation levels of CPGs. (F) Differences in methylation levels of CPGs. CPG: cuproptosis-promoting gene. [file Image_1.tif]

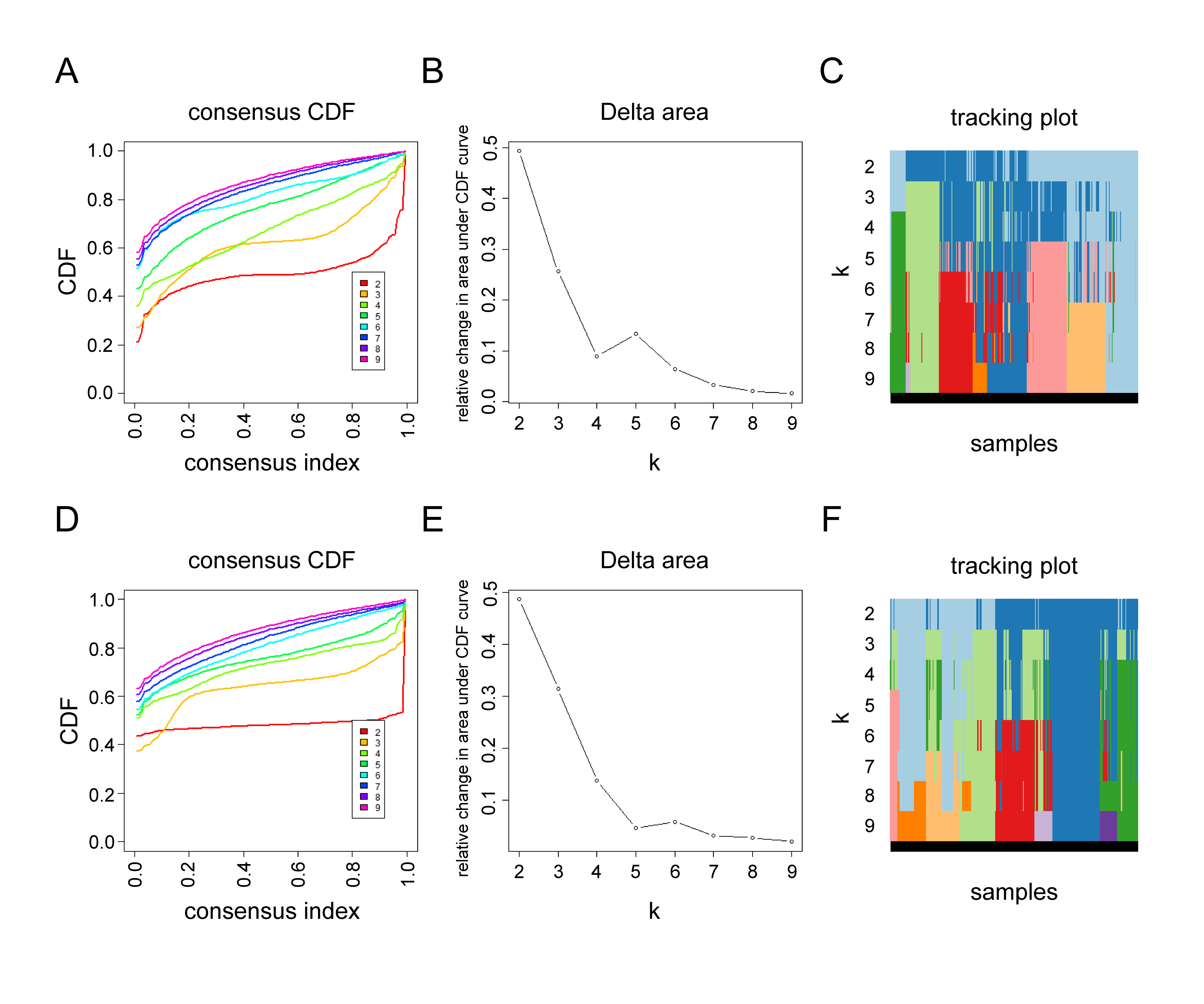

Supplement: Supplementary Figure 2 — Detailed results of consensus clustering. (A–C) Detailed Results of consensus clustering to construct CPGs subtypes: cumulative distribution curve (A), area under the cumulative distribution curve (B) and tracking plot (C). (D–F) Detailed Results of consensus clustering to construct CSRGs gene subtypes: cumulative distribution curve (D), area under the cumulative distribution curve (E) and tracking plot (F). CPG, cuproptosis-promoting gene; CSRG, cuproptosis subtypes related gene. [file Image_2.tif]

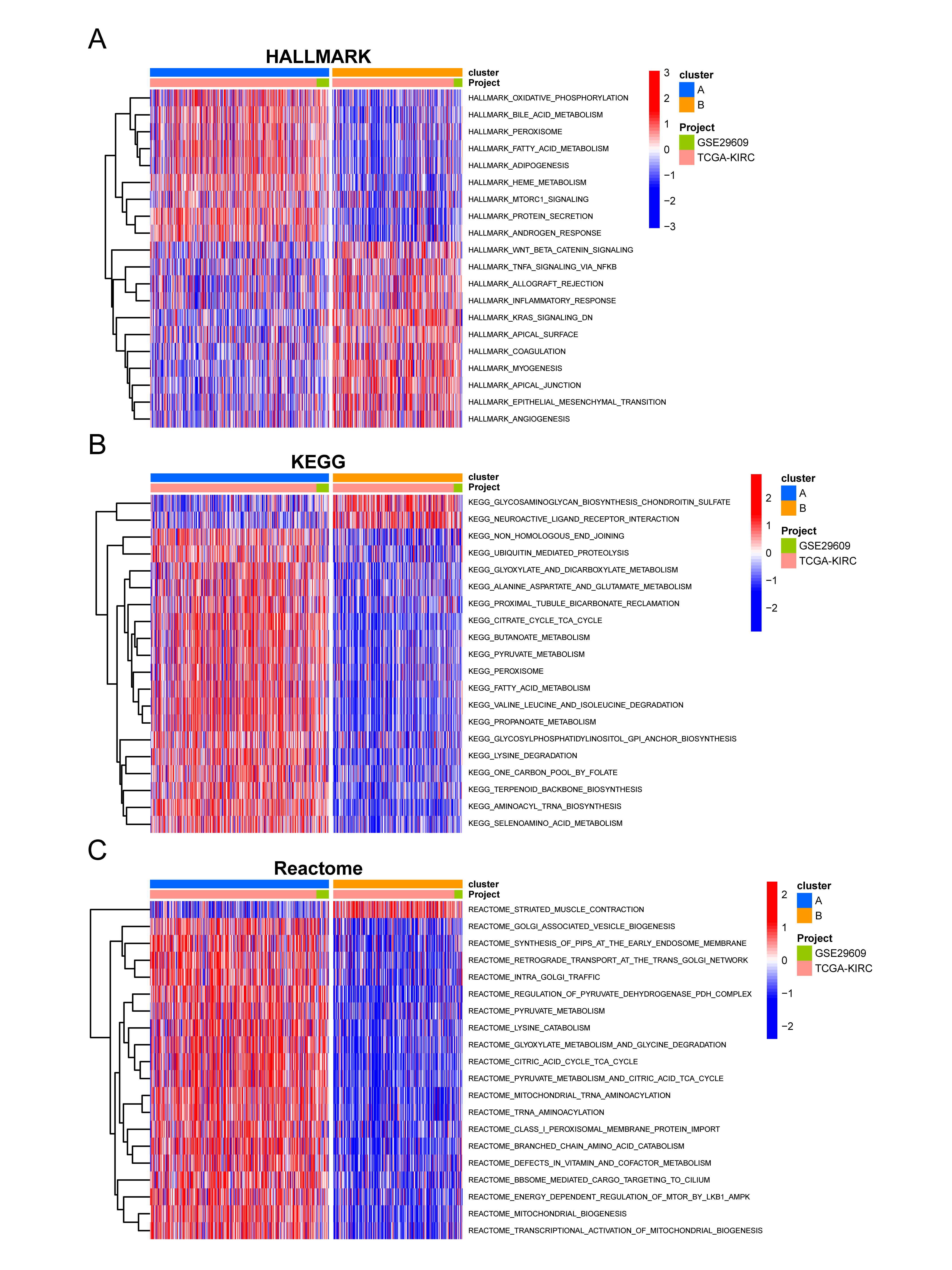

Supplement: Supplementary Figure 3 — Difference comparison of enriched pathways of two different CPG subtypes by GSVA. (A–C) Heatmaps comparing GSVA pathway scores for two CPG subtypes from three items: HALLMARK (A), KEGG (B), and Reactome (C). CPG, cuproptosis-promoting gene. [file Image_3.tif]

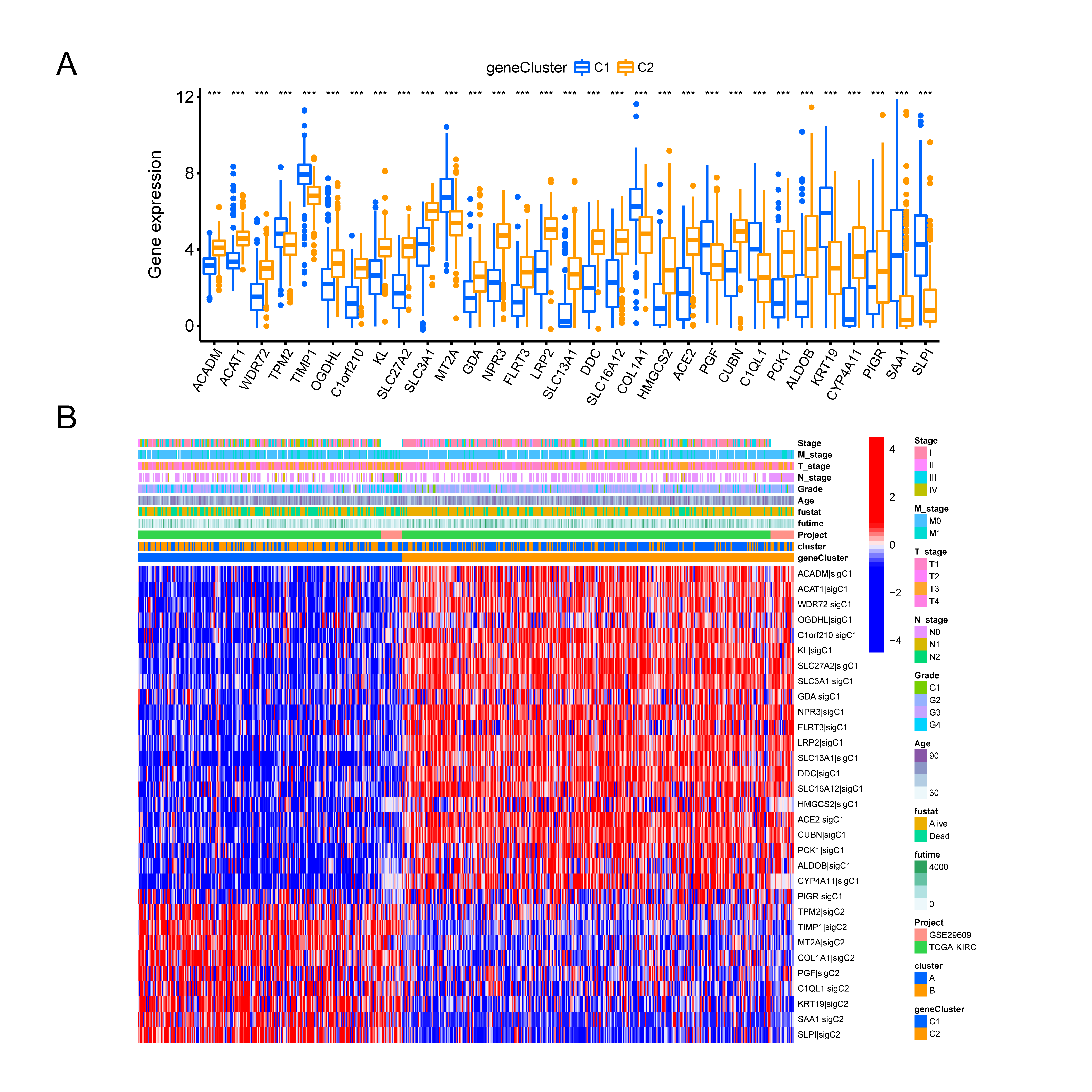

Supplement: Supplementary Figure 4 — Differences in clinicopathological and biological features between two gene subtypes. (A) Expression differences of 31 CSRGs between the two gene subtypes. (B) Heatmap of the distribution of clinicopathological features and CSRG expression between two different gene subtypes. CSRG, cuproptosis subtypes related gene; ***p < 0.001. [file Image_4.tif]

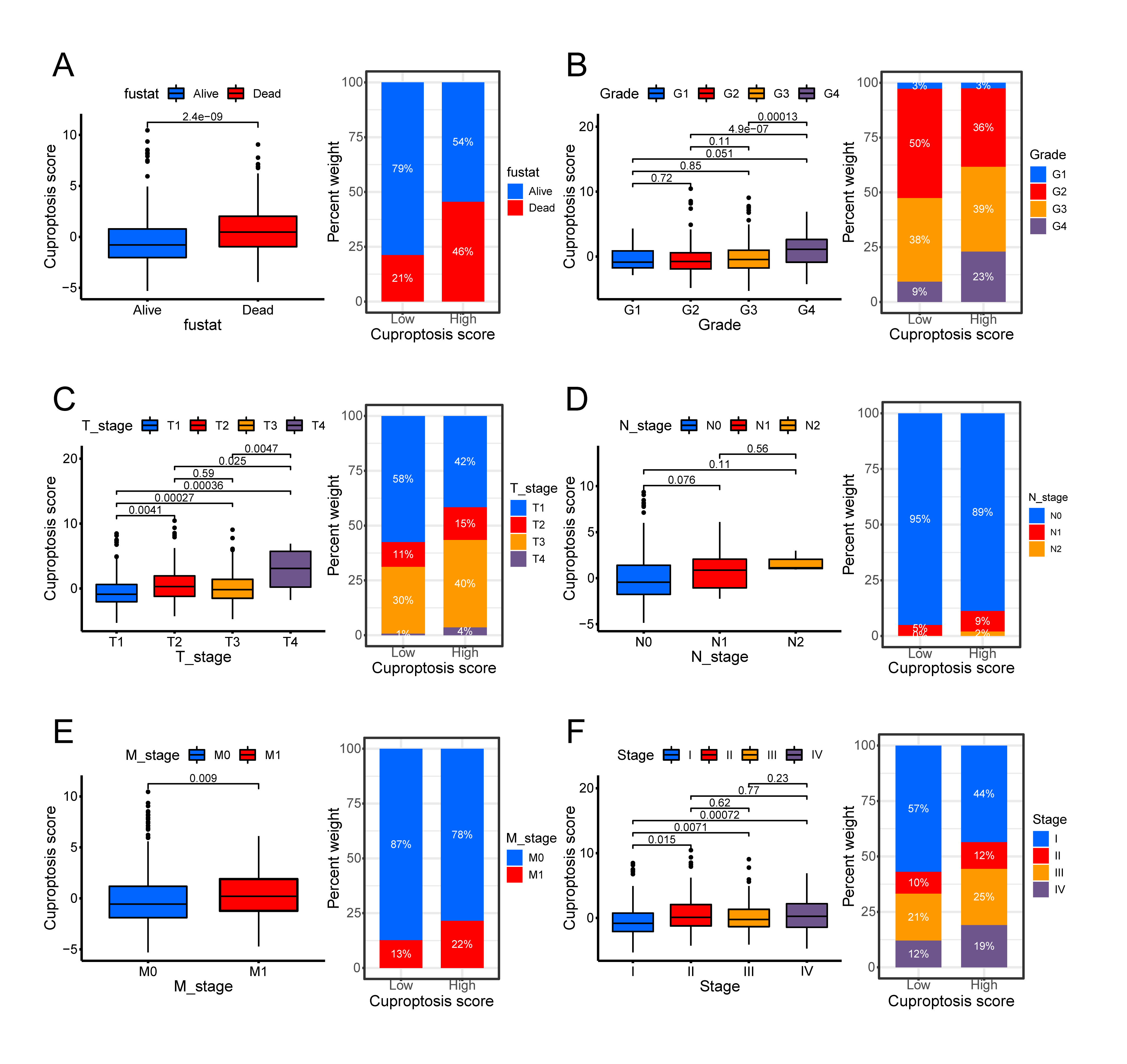

Supplement: Supplementary Figure 5 — Clinical subgroup analysis of CUS in KIRC. (A–G) Finding the relationship between six clinical features and CUS by difference comparison and ratio distribution: fustat (A), Grade (B), T_stage (C), N_stage (D), M_stage (E) and Stage (F). CUS, cuproptosis score. [file Image_5.tif]

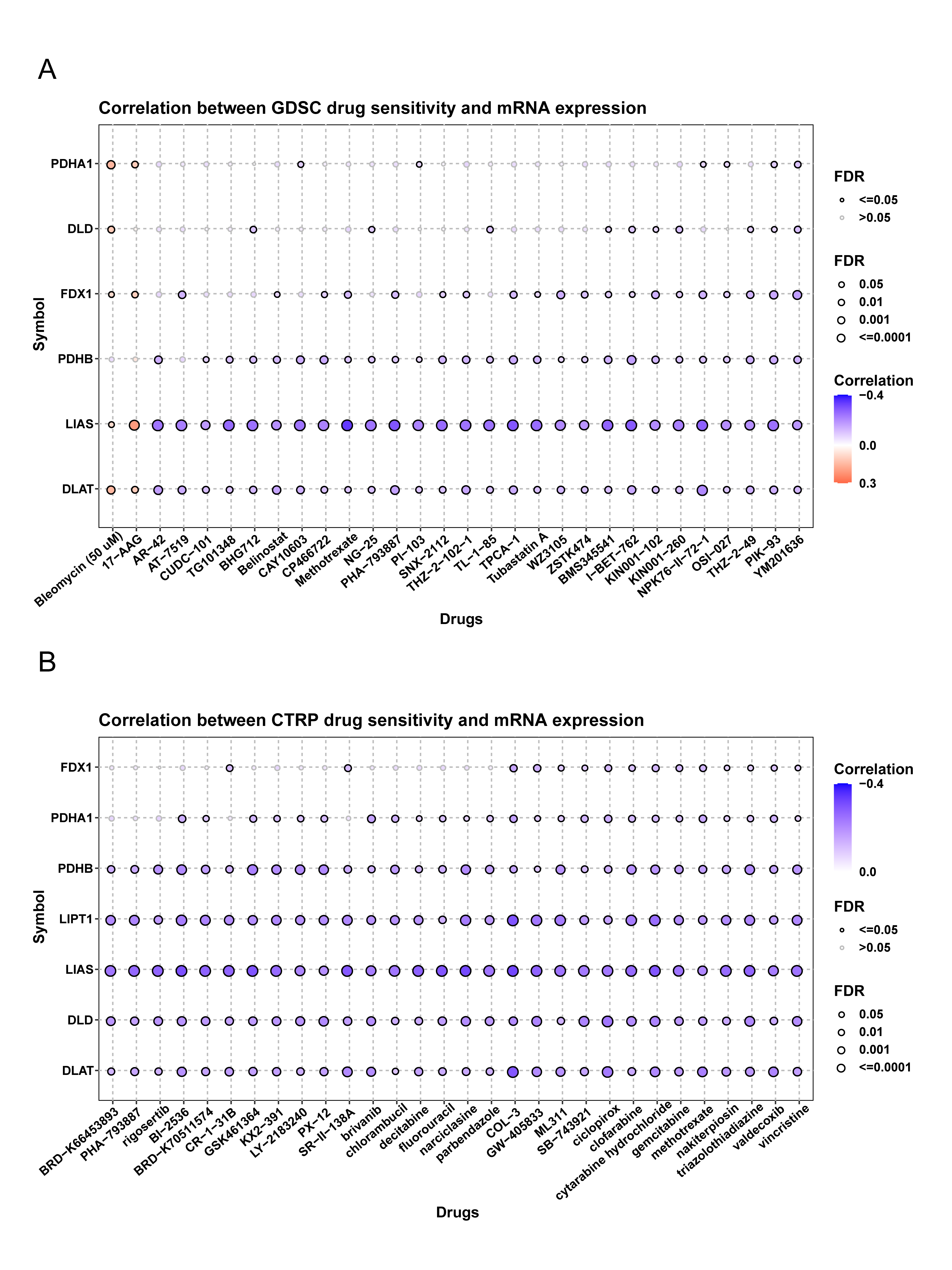

Supplement: Supplementary Figure 6 — Observation of the sensitivity of CPGs to targeted drugs using the GSCA database. (A) Correlation between GDSC drug sensitivity and CPGs mRNA expression. (B) Correlation between CTRP drug sensitivity and CPGs mRNA expression. CPG, cuproptosis-promoting gene. [file Image_6.tif]
