# Supplementary material for: Cuproptosis status affects treatment options about immunotherapy and targeted therapy for patients with kidney renal clear cell carcinoma
Source: Front Immunol. 2022 Aug 19;13:954440. doi: 10.3389/fimmu.2022.954440 (PMC9437301; doi:10.3389/fimmu.2022.954440)
Supplement: Supplementary file 7 [file Table_1.docx]

**Supplementary Table 1 |** Univariate COX regression analysis of 31 differential genes in cuproptosis typing.

| Gene Symbol | HR (95%CI) | P.value |
| --- | --- | --- |
| ACADM | 0.541 (0.458-0.639) | 4.94E-13 |
| ACAT1 | 0.590 (0.503-0.692) | 7.58E-11 |
| WDR72 | 0.606 (0.534-0.687) | 5.70E-15 |
| TPM2 | 1.302 (1.136-1.494) | 0.000159 |
| TIMP1 | 1.574 (1.376-1.802) | 4.36E-11 |
| OGDHL | 0.760 (0.679-0.850) | 1.48E-06 |
| C1orf210 | 0.690 (0.617-0.772) | 1.01E-10 |
| KL | 0.710 (0.642-0.785) | 1.98E-11 |
| SLC27A2 | 0.731 (0.667-0.800) | 1.50E-11 |
| SLC3A1 | 0.738 (0.680-0.801) | 2.58E-13 |
| MT2A | 1.295 (1.167-1.436) | 1.05E-06 |
| GDA | 0.761 (0.676-0.857) | 6.52E-06 |
| NPR3 | 0.729 (0.669-0.794) | 3.50E-13 |
| FLRT3 | 0.726 (0.649-0.813) | 2.44E-08 |
| LRP2 | 0.797 (0.740-0.858) | 1.89E-09 |
| SLC13A1 | 0.805 (0.733-0.885) | 6.78E-06 |
| DDC | 0.787 (0.726-0.853) | 4.93E-09 |
| SLC16A12 | 0.700 (0.644-0.760) | 1.89E-17 |
| COL1A1 | 1.219 (1.115-1.332) | 1.31E-05 |
| HMGCS2 | 0.820 (0.755-0.890) | 2.08E-06 |
| ACE2 | 0.785 (0.728-0.847) | 3.28E-10 |
| PGF | 1.114 (1.019-1.219) | 0.017917 |
| CUBN | 0.776 (0.720-0.836) | 2.85E-11 |
| C1QL1 | 1.133 (1.052-1.220) | 0.000913 |
| PCK1 | 0.804 (0.742-0.871) | 8.82E-08 |
| ALDOB | 0.856 (0.800-0.915) | 5.74E-06 |
| KRT19 | 1.110 (1.047-1.177) | 0.000456 |
| CYP4A11 | 0.847 (0.788-0.909) | 5.28E-06 |
| PIGR | 0.936 (0.877-0.999) | 0.047316 |
| SAA1 | 1.156 (1.106-1.209) | 1.19E-10 |
| SLPI | 1.148 (1.090-1.210) | 2.26E-07 |
