# Supplementary material for: Cuproptosis status affects treatment options about immunotherapy and targeted therapy for patients with kidney renal clear cell carcinoma
Source: Front Immunol. 2022 Aug 19;13:954440. doi: 10.3389/fimmu.2022.954440 (PMC9437301; doi:10.3389/fimmu.2022.954440)
Supplement: Supplementary file 8 [file Table_2.docx]

**Supplementary Table 2** **|** Information of primer sequence in qRT-PCR and siRNA sequence.

| Gene Symbol | NCBI ID | Sequence（5' to 3'） | |
| --- | --- | --- | --- |
| FDX1 | 2230 | F | GCCATTCTCTCACGCAAGGAT |
|  |  | R | ACGTGGTAATCTGTGGTGCTT |
| DLAT | 1737 | F | GCGACGGGCTCAGAATGTAG |
|  |  | R | CGCCATACCCTGTAGTCACG |
| DLD | 1738 | F | TTGGCAAAGACTTGGTGCAG |
|  |  | R | CCCTGTTTTTGAAGGATGCGT |
| LIAS | 11019 | F | TGATCCTCGGGCCAATTTTGAT |
|  |  | R | CATCATTCTCGCCTAAACCCAAC |
| LIPT1 | 51601 | F | ACGGTTGATCCCGAACACAG |
|  |  | R | ACGGTCGTGTGCATGTTTTAC |
| PDHA1 | 5160 | F | GACTCGGGAACAAGAAGGCA |
|  |  | R | TTAATGCCTGGCTGGGAGTG |
| PDHB | 5162 | F | GTTGCGGCCCATTTGTGAAT |
|  |  | R | ATACCAGGCAGCAAAGCACT |
| ACADM | 34 | F | GGGAGGTTGATTCTGGTCGTT |
|  |  | R | CATTGCCTCCAAGTATCTGCAC |
| ACAT1 | 38 | F | CTGGAGCCAGGATTGTTGGT |
|  |  | R | GCAGGCCTTCTGGTCACATT |
| si-FDX1#1 |  | F | GUGAUUCUCUGCUAGAUGUTT |
|  |  | R | ACAUCUAGCAGAGAAUCACTT |
| si-FDX1#2 |  | F | CUAACAGACAGAUCACGGUTT |
|  |  | R | ACCGUGAUCUGUCUGUUAGTT |
| si-FDX1#3 |  | F | GAUUAAAUGAGAACAUGGATT |
|  |  | R | UCCAUGUUCUCAUUUAAUCTT |

Notes: F: Forward; R: Reverse.
